# Spatiotemporal trends and ecological determinants of cardiovascular mortality among 2844 counties in mainland China, 2006–2020: a Bayesian modeling study of national mortality registries

**DOI:** 10.1186/s12916-022-02613-9

**Published:** 2022-11-30

**Authors:** Wei Wang, Junming Li, Yunning Liu, Pengpeng Ye, Chengdong Xu, Peng Yin, Jiangmei Liu, Jinlei Qi, Jinling You, Lin Lin, Ziwei Song, Limin Wang, Lijun Wang, Yong Huo, Maigeng Zhou

**Affiliations:** 1 National Center for Chronic and Noncommunicable Disease Control and Prevention, Chinese Center for Disease Control and Prevention, No. 27 Nanwei Road, Xicheng District, Beijing, 100050 China; 2 School of Statistics, Shanxi University of Finance and Economics, Taiyuan, Shanxi China; 3 The George Institute for Global Health, Faculty of Medicine and Health, University of New South Wales, Sydney, Australia; 4 Institute of Geographic Sciences and Natural Resources Research, Chinese Academy of Sciences, Beijing, China; 5 Department of Cardiology, Peking University First Hospital, No. 8 Xishiku Street, Xicheng District, Beijing, 100034 China

**Keywords:** Cardiovascular mortality, China, Spatiotemporal characteristics, nonmedical ecological determinants, Hierarchical Bayesian model, Population-based strategy

## Abstract

**Background:**

Cardiovascular disease (CVD) is the leading cause of death in China. No previous study has reported CVD mortality at county-level, and little was known about the nonmedical ecological factors of CVD mortality at such small scale in mainland China. Understanding the spatiotemporal variations of CVD mortality and examining its nonmedical ecological factors would be of great importance to tailor local public health policies.

**Methods:**

By using national mortality registration data in China, this study used hierarchical spatiotemporal Bayesian model to demonstrate spatiotemporal distribution of CVD mortality in 2844 counties during 2006 to 2020 and investigate how nonmedical ecological determinants have affected CVD mortality inequities from the spatial perspectives.

**Results:**

During 2006–2020, the age-standardized mortality rate (ASMR) of CVD decreased from 284.77 per 100,000 in 2006 to 241.34 per 100,000 in 2020. Among 2844 counties, 1144 (40.22%) were hot spots counties with a higher CVD mortality risk compared to the national average and located mostly in northeast, north central, and westernmost regions; on the contrary, 1551 (54.53%) were cold spots counties and located mostly in south and southeast coastal counties. CVD mortality risk decreased from 2006 to 2020 was larger in counties where CVD mortality rate had been higher in 2006 in most of the counties, vice versa. Nationwide, nighttime light intensity (NTL) was the major influencing factor of CVD mortality, a higher NTL appeared to be negatively associated with a lower CVD mortality, with one unit increase in NTL, and the CVD mortality risk will decrease 11% (relative risk of NTL was estimated as 0.89 with 95% confidence interval of 0.83–0.94).

**Conclusions:**

Substantial between-county discrepancies of CVD mortality distribution were observed during past 15 years in mainland China. Nonmedical ecological determinants were estimated to significantly explain the overall and local spatiotemporal patterns of this CVD mortality risk. Targeted considerations are needed to integrate primary care with clinical care through intensifying further strategies to narrow unequally distribution of CVD mortality at local scale. The approach to county-level analysis with small area models has the potential to provide novel insights into Chinese disease-specific mortality burden.

**Supplementary Information:**

The online version contains supplementary material available at 10.1186/s12916-022-02613-9.

## Background

Cardiovascular disease (CVD) remains the largest single contributor to global mortality and the leading cause of death (COD) in China that accounts for 40% of total deaths [1–3]. In comparison with other countries, CVD mortality burden in China was higher than the global average, far exceeded upper-income counties (such as USA), and was higher than those of upper-middle-income counties (such as UK, Australia, Japan and France) as well, located in the middle of G20 [4, 5]. During recent years, the State Council have endorsed series of important documents to reduce CVD mortality and promote CVD health, such as Health China 2030 Guidelines [6], 13th Five-Year Plan for Hygiene and Health, and Medium- to Long-Term Plan for the Prevention and Treatment of Chronic Diseases (2017-2025) [7]. Correspondingly, clarifying the CVD burden over time and space will help to consolidate government-dominant strategies to reduce the risk of CVD nationwide [8].

Mortality rates for small-area of the country such as counties could be able to differ substantially from the national average; thus, county-level spatial variations and trend patterns of mortality have important implications to inform local health policies [9, 10]. Internationally, previous studies have reported large-scale and long-standing all-cause and cause-specific mortality at county-level or at higher resolution to demonstrate increasing geographical disparities nationwide, like US and UK [9–14]. For example, Roth demonstrated trends and patterns of major COD and cardiovascular mortality at county-level in USA during 1980 to 2014, they found there were large between-county differences for each cause of CVD mortality, and they suggested that there was a need for pro-equity economic and social policies and greater investment in public health and health care throughout the entire country [9]. In Brazil, Baptista showed the spatiotemporal evolution of major cause of death by using data from the System Data Mortality Information from 1998 to 2017 across micro-regions, and substantial variations were observed between areas and sex, and those diversities was marked by large socioeconomic differences [14].

As the most populous country worldwide, China has a spatially stratified heterogeneity in nonmedical ecological determinants, such as medical technology, economy, culture, and geographic environment [1, 15]. These disparities affect overall CVD mortality, and sometimes, the influence of these factors far exceed the medical treatment itself, but their roles are often overlooked [15]. Effective intervention to reduce CVD mortality thus requires a better understanding of the exact geographical distribution and its impacts on CVD mortality [15, 16]. Determinants of health are the conditions in the environments where people are born, live, learn, work, play, worship, and age that affect a wide range of health, functioning, and quality-of-life outcomes and risks and they embody the consequences of multifaceted societal processes and norms that shape living conditions and produce a broad range of health disparities, which are roughly included medical and nonmedical ones [15, 17–19]. Among which, nonmedical factors often include socioeconomic, population or environmental factors which do not directly influence population health; while medical factors usually include elements that directly influence human health such as the pathways of medical prevention, diagnosis, treatment, or interventions [15, 17–19]. However, to our knowledge, existed studies in China merely reported CVD mortality distribution by administrative units such as nation, province, or among specific population [1, 8, 16]. Besides, litter domestic studies at county-level have neither attempted to depict spatiotemporal distribution of CVD mortality nor provided evidence to reveal the impacts of nonmedical ecological determinants on CVD mortality, which may mask variations in CVD mortality patterns among local communities and limit the ability to prioritize, plan for, and evaluate public health interventions.

Evidence-based planning and evaluation with a local focus is particularly important for CVD mortality since coverage and effectiveness of preventive interventions and treatments may vary locally [13]. Therefore, in order to reveal the knowledge gap, by using data from national mortality registries in mainland China, on the basis of consistent and comparable estimates, this study aimed to demonstrate spatiotemporal variations of CVD mortality risk at county-level and, also, to quantify the magnitude of how selected nonmedical ecological determinants have affected CVD mortality inequities from the spatial perspectives. It would further facilitate local information through demonstrate how routinely collected mortality data can be used to inform the inequities of local CVD mortality risk.

## Methods

### Data source

#### Mortality, under-reporting, and population data

Death certificate is regarded as an essential data source to measure and inform trends and patterns of disease mortality in public health practice [20]. This study used deidentified death cases collected by National Mortality Registration Information Management System (NMRIMS) housed in Chinese Center for Disease Control and Prevention (China CDC). Currently, National Mortality Surveillance System (NMSS) is the most populous mortality surveillance system worldwide and the only one with provincial representativeness in China, covers over 300 million individuals from 605 surveillance points (among which, “districts” was defined as urban areas and “counties” was defined as rural areas) in 31 provincial-level administrative divisions (PLADs, excluding Hong Kong Special Administrative Region, Macau Special Administrative Region and Taiwan province) in mainland China that accounts for 24% of total population and routinely collects individual details of death information in real-time through an internet-based approach [21]. Other than surveillance points included in NMSS, those rest of districts/counties also reported death cases directly through NMRIMS to China CDC; thus, NMRIMS have collected death cases from nearly all districts/counties across the country. Apart from national mortality registries, data for under-reporting adjustment were obtained from NMSS under-reporting field surveys conducted in 2009, 2012, 2015, and 2018, separately, which collected under-reporting data from 2006–2017 [22]. Data for county-level population was obtained from National Bureau of Statistics of China [23]. Data for CVD mortality rate standardization was acquired from 2010 population census [23]. Detailed descriptions of data source for NMSS, NMRIMS, under-reporting surveys for national mortality registries, population data, and 2010 Chinese census accompanied with their data curation process were reported in the additional file (see Additional file 1: Part 1. Table S1, Table S2).

#### Nonmedical ecological determinants

On the basis of the ecological model of health determinants developed by Dahlgren and Whitehead, the health determinants of focused outcomes could be attributed to direct causes (or proximal factors, downstream determinants) like disease-specific causes or individual risk factors and indirect causes (or distant factors, upstream determinants) like social determinants or environment factors [24]. In this study, we do not analyze the medical causes but rather focus on nonmedical ecological determinants at county-level [17, 18]. For which, being the upstream capstone of social development and preconditions of other factors, nonmedical ecological determinants play much important role in tailoring public health practice in real world, especially at population level, among either CVD patients, population at high risk, or even normal people [1, 25–27].

One of the most important nonmedical ecological determinants, also called indirect determinants, was gross domestic product per capita (GDP, 10,000 yuan per person), and it reflected to each resident’s economic contribution or value creation of his country or region [1, 26–28]. Second is nighttime light (NTL) data, which is often used to involve human social activities and urban expansion, socioeconomic factors estimation, and other fields such as environment, disaster, fishery, and energy [29]. Third, we included number of beds in health care institutions (NB, units per 10,000 persons) to present local healthcare resources and capacity [23]. Fourth, we included population density (PD, persons per 1 square kilometer) to present the population distribution in a specific area [23]. Apartment from that, we summarized that both the environmental and meteorological factors like particulate matters emission, temperature, and humidity, might have impacts on CVD mortality [30–32]. We thus included annual average temperature (TEMP, °C), temperature variability (TV, °C), annual average relative humidity (HUMID, %), longitude (LT), altitude (AT), and concentration of PM_2.5_ emission (PM2.5, *μg*/*m*^3^), as confounders in main analysis. We utilized multiple imputation to deal with the missing data of each determinant. Detailed descriptions of those indicators and confounders were reported in the additional file (see Additional file 1: Part 1. Table S3).

### Participants

#### Under-reporting adjustment and all-cause mortality calculation

In order to ensure the reliability and validity of data from NMRIMS, we calculated under-reporting rate (URR) annually for each stratum (urban/rural areas in eastern/central/western regions) during 2006–2017 as the proportion of missed deaths among the total number of deaths identified in under-reporting surveys. We then used spline regressions to predict URR in each stratum during 2018–2020. Afterwards, we derived under-reporting-adjusted all-cause mortality rate by sex and age group for all districts/counties by dividing reported number of deaths by (1-URR) [16].

#### Data quality control

In order to acquire consistent information of mortality at district/county level, we first unified administrative codes throughout 2006-2020 to an identical one to deal with the issues caused by newly-added, cancelation, alternation of the codes in each of the district/county. Afterwards, we have reviewed, compared, and evaluated the data quality of each county to exclude certain ones that were considered to be seriously under-reported and might affect overall results. We made the under-reporting-adjusted all-cause mortality rate of lower than 4.5‰ as exclusion criteria for those counties belongs to VRs and DSPs earlier than 2013 [33]. Since 2013, we made the criteria of 5‰. For counties that neither belongs to VRs nor DSPs, we made the criteria of 3‰ during 2006–2020. Afterwards, we performed a covariate-based modeling approach to further evaluate the data quality of remained counties [33]. We also excluded death cases with missing values of key indicators like COD, sex, and age. Detailed descriptions of data quality control were reported in the additional file (see Additional file 1: Part 2).

#### CVD mortality calculation

Underlying COD in NMRIMS was recorded by using International Classification of Disease 10th Edition (ICD-10). All death cases between 2006 and 2020 where CVD was identified as underlying COD were extracted (ICD10: I00-I99) [33]. CVD mortality rate by location-year-sex-age group was calculated by multiplying by all-cause mortality rate generated previously and proportion of COD, for which the latter was calculated by cases of CVD deaths divided by all-cause deaths at each stratum [16].

### Statistical methods

#### HBSTM framework

We applied hierarchical Bayesian spatiotemporal model (HBSTM) to reveal the spatiotemporal patterns and measure the associations between nonmedical ecological determinants of CVD mortality at county-level in mainland China during 2006–2020. Comparing with classical regression models, by taking a spatially explicit approach to modeling, the spatial configuration of the data may contribute information about the outcome not captured by other area attributes. Moreover, HBSTM is proposed in the form of prior knowledge incorporated with spatial and temporal correlations and uncertainties existed in spatiotemporal process, which could fully depict spatiotemporal evolution of disease distribution. HBSTM framework consists of three models: data model, process model, and (hyper) parameter model, and it can be described as follows [15, 34–44]:

1 $$\textrm{Data}\ \textrm{model}:\kern0.5em {\textrm{Y}}_{it}\sim P\left({\textrm{Y}}_{it}|{\theta}_{it},\Theta \right)$$

2 $${\displaystyle \begin{array}{cc}\textrm{Process}\ \textrm{model}:& {\theta}_{it}=\textrm{S}(i)+\omega (t)+{\Omega}_{it}\left(i,t\right)+\end{array}}{\varepsilon}_{it}$$

3 $${\displaystyle \begin{array}{cc}\left(\textrm{Hyper}\right)\ \textrm{Parameter}\ \textrm{model}:& \Theta \sim \textrm{P}\end{array}}\left(\Theta \right)$$

In Eqs. (1), (2), and (3), *i* denotes a certain county in China; *t* denotes a specific year during 2006 and 2020; *Y*_*it*_ denotes the observed sample data with spatiotemporal attributes; *θ*_*it*_ denotes the spatiotemporal process metric; S(*i*) denotes the overall spatial pattern; *ω*(*t*) denotes the overall temporal trend; Ω_*it*_(*i*, *t*) denotes the local spatiotemporal effects; *ε*_*it*_ denotes the spatiotemporal stochastic noise; and Θ denotes the (hyper) parameter data.

#### HBSTM specification

For modeling count data of rare events like CVD mortality occurred in a small-scale area with large population, Poisson regression is often applied, and it can be specified as a log-linear model for the CVD mortality as follows [15, 34–36, 38–46]:

4 $${Y}_{it}\sim \textrm{Poisson}\left({E}_{it}{\theta}_{it}\right)$$

In Eq. (4), *Y*_*it*_ denotes the observed CVD death counts in county *i* and year *t*, and it follows a Poisson distribution. *E*_*it*_ denotes the expected CVD death counts in county *i* and year *t*. *θ*_*it*_ denotes a county specific CVD mortality risk, which is equivalent in this setting to the standardized mortality ratio (SMR), as the SMR is often seen as a highly unstable outcome because of the number of events and the size of population at risk, and this instability needs to be accounted for in the model. By using methods of hierarchical Bayesian smoothing, this can be addressed directly and specified as follows [15, 34, 36–46]:

5 $${\displaystyle \begin{array}{c}\mathit{\log}\left({\theta}_{it}\right)=\alpha +{S}_i+{V}_t+{\varphi}_{it}\\ {}\mathit{\log}\left({\theta}_{it}\right)=\alpha +{s}_i+{u}_i+{b}_0t+{v}_t+{b}_{1i}\textrm{t}+{\varepsilon}_{it}\end{array}}$$

6 $$\mathit{\log}\left({\theta}_{it}\right)=\alpha +{s}_i+{u}_i+{b}_0t+{v}_t+{b}_{1i}\textrm{t}+{\varepsilon}_{it}+\beta X$$

In Eq. (5), the observed spatiotemporal variability in CVD mortality risk is decomposed into three components: overall spatial pattern *S*_*i*_(*s*_*i*_ + *u*_*i*_), overall temporal trend *V*_*t*_(*b*_0_*t* + *v*_*t*_), and local spatiotemporal variations *φ*_*it*_ (*b*_1*i*_t + *ε*_*it*_) within each spatial unit (district/county). Among which, *α* denotes the intercept quantifying the average CVD mortality rate in all counties in China over 2006–2020. For spatial terms, *s*_*i*_ denotes the spatially structured random effects and is used to describe the distribution of CVD mortality risk across China during the study period, and *u*_*i*_ denotes the spatially unstructured random effects. The overall time trend consists of a linear temporal trend *b*_0_, which represents the overall rate of change in CVD mortality and is also called overall slope, with an additional Gaussian noise *v*_*t*_ used to detect nonlinear trend. The combination of the common spatial pattern and temporal trend represents the stable component of CVD mortality risk. Apart from this, local spatiotemporal variations represent the spatial and temporal interaction effects describing local attributes, among which, *b*_1*i*_ denotes the departure from global trend for each county and also called local slope, *ε*_*it*_ denotes the spatiotemporal stochastic noise for county *i* in year *t*, and captures additional variability that not explained by other terms in the model. In this study, we used various random effects to partition the observed space-time variations, and those random effects serve as surrogate measures of unobserved risk factors that vary over space, time or both. Further, we extended null spatiotemporal model, Eq. (6), to incorporate covariates that could help to explain the spatiotemporal patterns, where *X* represents a series of covariates and *β* denotes their corresponding coefficients. In this study, we included GDP, NTL, NB, and PD as potential influential factors, and TEMP, TV, HUMID, LT, AT, and PM2.5 as confounders.

Prior and hyperprior were assigned for all parameters in the model [16, 34, 36–46]. For prior distribution specifications, we assumed that the overall CVD mortality risk *α* and overall rate of CVD mortality risk change *b*_0_ followed a uniform distribution. We assumed that the overall spatial random effect *s*_*i*_ and local departure from global trend *b*_1*i*_ followed a conditional autoregressive (CAR) prior with a spatial weight matrix *W* to impose spatial structure. Specifically, *W* is a matrix of size *N* × *N*, where its diagonal entries *w*_*ii*_ = 0 and the off-diagonal entries *w*_*ij*_ = 1 if county *i* and *j* share a common boundary and *w*_*ij*_ = 1 otherwise. Herein, CAR prior on the spatial random effect implies that the adjacent counties tended to have changes in CVD mortality risk that were more alike than is the case for counties that were far apart. Both of the nonlinear temporal trends *v*_*t*_ and overdispersion parameter *ε*_*it*_ followed a normal distribution. For hyperprior distribution specifications, we assigned a strictly positive half Gaussian prior to all random effect standard deviations, and precision parameter followed gamma noninformative distribution. Detailed descriptions of prior and hyperprior parameter specifications and model selection were reported in the additional file (see Additional file 1: Part 3).

Afterwards, we visualized the spatial variations of CVD mortality risk and its change at county-level through using the results of SMR of CVD estimated by HBSTM. In the consideration of soaring death cases reported by the national mortality registries especially after its expansion in 2013, as well as the population structure change, we calculated absolute value of age-standardized mortality rate (ASMR) of CVD to provide a comparable estimation across time and space. Thereby we used average annual percent change (AAPC) to present ASMR of CVD at county-level that change at a constant percentage every year change linearly on a log scale. AAPC is a summary measure of the trend over a pre-specified fixed interval, and it allows us to use a single number to describe the average percent change over a period of multiple years [47].

#### CVD mortality risk classification

We also reported the posterior probability which estimated the relative change over time and space in each county to represent an increase versus a decrease of CVD mortality risk. The posterior probability represented the inherent uncertainty of changing CVD mortality trends, specifically, if the estimated CVD mortality was the same in 2006 and 2020, and an increase was statistically indistinguishable from a decrease, there was a 50% posterior probability of an increase and a 50% posterior probability of a decrease. For example, in a county in which the posterior distribution of CVD mortality in 2020 was entirely greater than in 2006, there was around a 100% posterior probability of an increase, and hence around a 0% probability of a decrease, and vice versa. Posterior probabilities more distant from 50% indicate more certainty, and it can be described as follows [12, 44]:

7 $$p\left(\mathit{\exp}\left({s}_i+{u}_i\right)>1| data\right)$$

8 $$p\left({b}_{1i}>0|{h}_i, data\right)$$

In Eqs. (7) and (8), (*exp*(*s*_*i*_ + *u*_*i*_)) denotes the logarithm of CVD mortality rate over time in county *i* relative to national average, *data* denotes the observed data, and *p*(*exp*(*s*_*i*_ + *u*_*i*_) > 1| *data*) denotes the posterior probability of relative risk of CVD mortality larger than 1. *p*(*b*_1*i*_ > 0| *h*_*i*_, *data*) denotes the posterior probability of local trend of CVD mortality risk in county *i* relative to national average larger than 0.

On the basis of this, we performed a two-stage classification method to identify hot/cold/warm spots of CVD mortality risk at county-level by using the *p*(*exp*(*s*_*i*_ + *u*_*i*_) > 1| *data*) and *p*(*b*_1*i*_ > 0| *h*_*i*_, *data* )[12, 15, 44]. For details, at the first stage, we defined a county as a hot spot (*h*_1_) if *p*(*exp*(*s*_*i*_ + *u*_*i*_) > 1| *data*) was greater than 0.8 and as a cold spot (*h*_2_) if *p*(*exp*(*s*_*i*_ + *u*_*i*_) > 1| *data*) was less than 0.2; besides, we defined the other counties as warm spots (*h*_3_). At the second stage, we further classified a county under each risk category in the first stage into one of the three trend patterns by using estimates of local slopes *b*_1*i*_, namely, a county with a faster increasing local trend than global trend if *p*(*b*_1*i*_ > 0| *h*_*i*_, *data*) > 0.8, a decreasing trend relative to the global trend if *p*(*b*_1*i*_ > 0| *h*_*i*_, *data*) < 0.2, and a local trend not differing from the global trend if 0.2 < *p*(*b*_1*i*_ > 0| *h*_*i*_, *data*) < 0. 8 [15, 34, 41, 44]. Detailed descriptions of CVD mortality risk classifications were reported in the additional file (see Additional file 1: Part 3. Table S4).

In this study, 95% confidence intervals (CI) were used to represent the 2.5th to 97.5th percentiles of the posterior distribution of estimated CVD mortality, and a *P* value < 0.05 was considered statistically significant; all tests were two sided. Data cleaning and preparation was performed in SAS version 9.4 (SAS Institute Inc., Cary, North Carolina, USA), while spatiotemporal modeling was performed in R version 4.1.2 (The R foundation for Statistical Computing, Lucent Technologies, Auckland, New Zealand) by using “INLA” package.

## Results

### Characteristics of CVD mortality nationwide

Generally, as shown in the additional file (see Additional file 1: Part 4. Table S5), during 2006–2020, the number of districts/counties that reported death cases to NMRIMS ranged from over 2600 to nearly 3100, with the highest number of 3065 in 2013 and lowest number of 2698 in 2006. We first acquired 2656 districts/counties with consistent administrative codes during the years, and 2108 districts/counties were left and included in the main analysis when covariates-based data quality process was performed. Between 2006 and 2020, it was observed that the CVD death increased from 266,331 cases to 3,333,311 cases with 27,100,877 cases in total in the analysis of the study.

### ASMR of CVD mortality at county-level

Overall, the estimated ASMR of CVD decreased from 286.94 per 100,000 in 2006 to 241.34 per 100,000 in 2020 nationwide. In 2020, urban areas (206.79 per 100,000), female population (202.00 per 100,000), and eastern regions (219.10 per 100,000) showed lower ASMR of CVD compared with their counterparts.

**Fig. 1:**
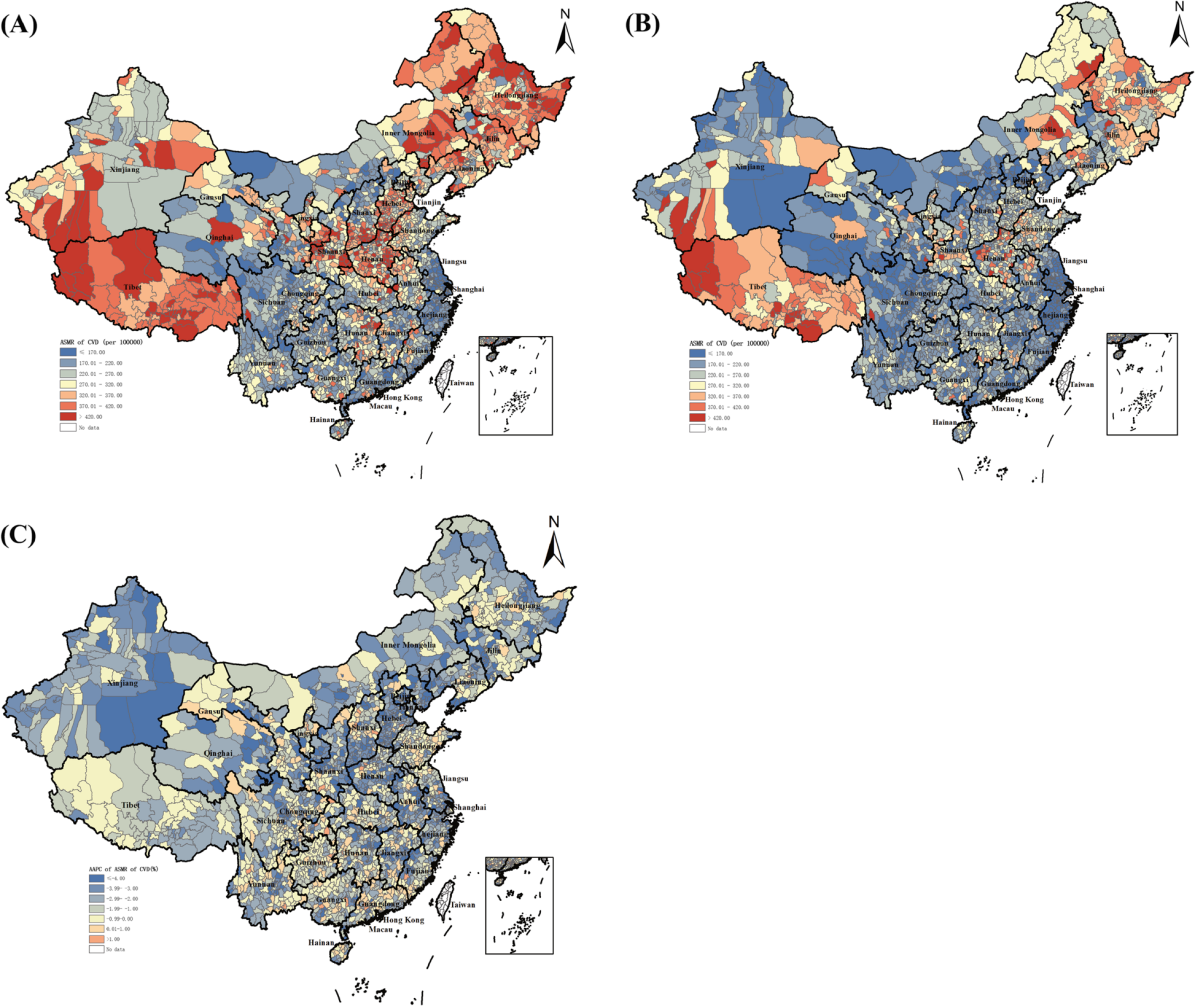
ASMR of CVD and its change at county-level in China, 2006–2020. **A** ASMR of CVD in 2006, per 100,000. **B** ASMR of CVD in 2020, per 100,000. **C** AAPC of ASMR of CVD during 2006–2020 (%)

Figure 1 shown the results of ASMR of CVD and its change estimated by HBSTM in 2844 counties in China. In 2006 and 2020, the ASMR of CVD presented similar spatial patterns among total and each of subpopulation among male and female CVD patients (see Additional file 1: Part 4. Fig. S1, Fig. S2). Specifically, Chinese counties located in northeast, north central, and southwest provinces experienced the highest CVD mortality, including Heilongjiang, Jilin, Liaoning, Hebei, Shanxi, Henan, and Tibet, among which, in 2020, Ritu county (479.19 per 100,000) in Tibet, Gaer county (475.47 per 100,000) in Tibet, and Nanshan county (474.35 per 100,000) in Heilongjiang were three leading counties with the highest CVD mortality. On the contrary, among most of counties with lower CVD mortality located in southeast and southern provinces like coastal areas Jiangsu, Zhejiang, Fujian, and Guangdong, in 2020, Rudong county (163.57 per 100,000) in Jiangsu, Xihu county (182.51 per 100,000) in Zhejiang, and Jiading county (191.20 per 100,000) in Shanghai were three counties with the lowest CVD mortality. On average, the change of ASMR of CVD mortality during 2006 and 2020 indicated a comparatively dispersed distribution across the country, with Zhuanglang county (49.58%) in Gansu, Jiayin county (47.42%) in Heilongjiang, and Baiyunkuang county (47.17 %) in Inner Mongolia experienced the highest percentage of decrease.

### Spatiotemporal distribution of CVD mortality at county-level during 2006-2020

**Fig. 2:**
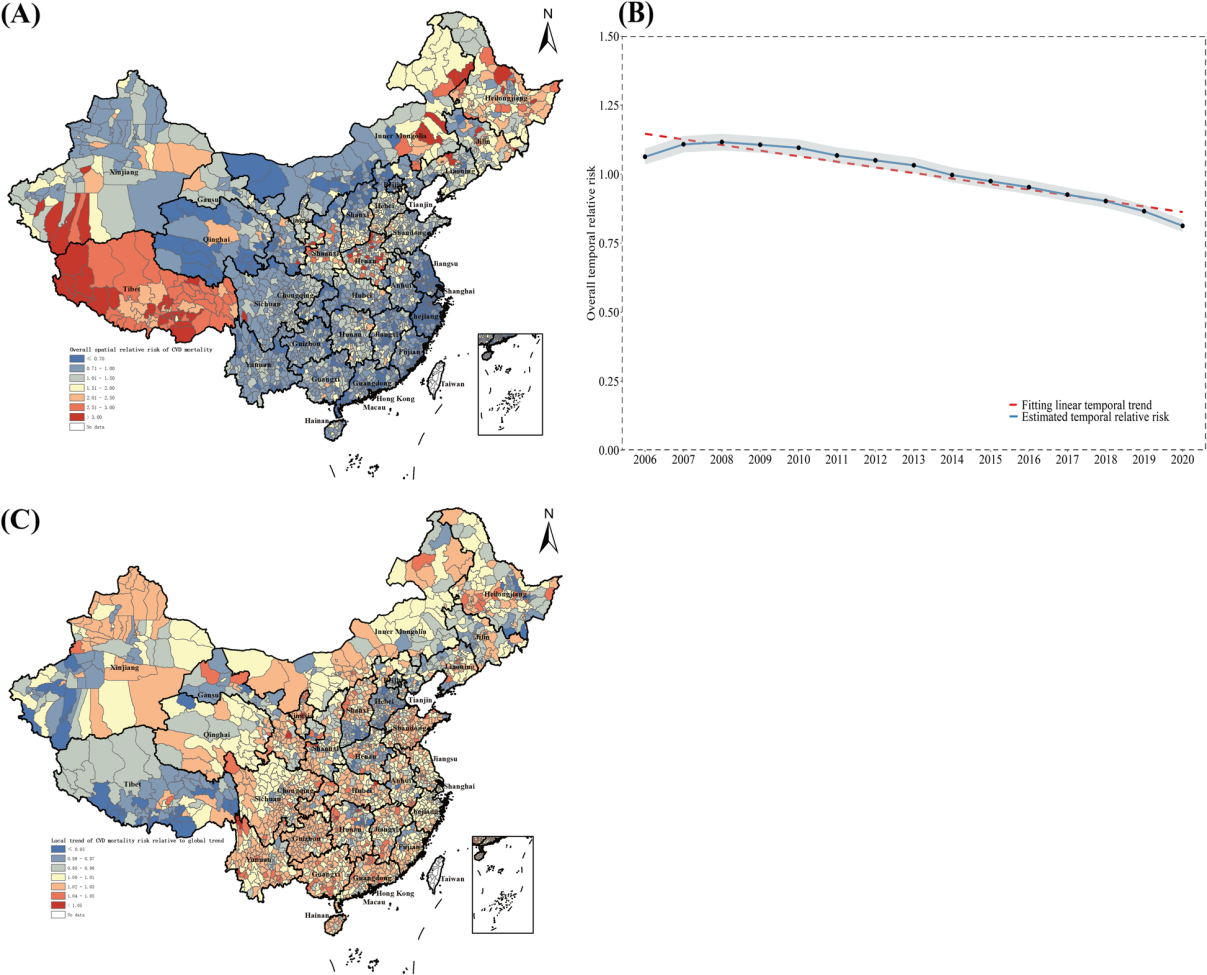
Spatiotemporal patterns of relative risk of CVD mortality at county-level in China, 2006-2020. A Posterior mean of the overall spatial relative risk (***exp***(***s***_***i***_ **+** ***u***_***i***_)) of CVD mortality, 2006–2020. B Posterior mean of the overall temporal relative risk (***exp***(***b***_**0**_***t*** **+** ***v***_***t***_)) with a probability of 95% of CVD mortality, 2006-2020. C Posterior mean of the local spatiotemporal trend (***exp***(***b***_**1*i***_)) of CVD mortality relative to global spatiotemporal trend, 2006–2020

Figure 2 shown the spatiotemporal distribution of CVD mortality risk at county-level during 2006–2020 across the country. As shown in Fig. 2A, we used posterior mean of the overall spatial relative risk (including spatial structured and unstructured random effects) of CVD mortality during 2006–2020 to demonstrate overall spatial patterns of CVD mortality at county-level. Generally, the spatial relative risk was estimated with substantially geographical diversities across the country with its posterior mean ranged from 0.36 to 7.28. Among which, the darker the warm colors were shaded, the higher CVD mortality risk was estimated, such as red, orange, and yellow; conversely, the darker the cold colors were shaded; the lower CVD mortality risk was estimated, such as blue. Specifically, districts/counties with the overall spatial relative risk (*exp*(*s*_*i*_ + *u*_*i*_) > 2.0) were regarded as areas with higher CVD mortality risk, and most of them located in northeast provinces like Heilongjiang, Jilin, and Liaoning, central provinces like Henan, and western provinces like Tibet. And, districts/counties with the overall spatial relative risk (*exp*(*s*_*i*_ + *u*_*i*_) > 1.0) were regarded as areas with lower CVD mortality risk, most of them located in southeast coastal provinces like Zhejiang, Shanghai, and Jiangsu. Besides, similar spatial relative risk of CVD mortality was observed in men and female population (see Additional file 1: Part 4. Fig. S3, Fig. S4). As shown in Fig. 2B, we used posterior mean of the overall temporal relative risk (including a linear and nonlinear temporal trends) of CVD mortality during 2006–2020 to demonstrate the overall temporal trend nationwide. Generally, CVD mortality risk was estimated to present a steady downward movement from 2006 to 2020, and the trend was much faster among female population compared with male population (see Additional file 1: Part 4. Fig. S3, Fig. S4). Additionally, as illustrated in Fig. 2C, we used posterior mean of the local trend of CVD mortality risk relative to global trend (including local slope and spatiotemporal stochastic variations) to demonstrate the departure of local spatiotemporal variations compared with global condition. For whole population, the local spatiotemporal trend was estimated with posterior mean ranged from 0.92 to 1.09. Among which, the darker the warm colors were shaded, the more substantial departure was estimated between local and national trend, while the darker the cold colors were shaded, the more identical spatiotemporal variations existed for local and national trend. Specifically, districts/counties with the local spatiotemporal trend of CVD mortality risk (*exp*(*b*_1*i*_) > 1.0) were regarded as areas having substantial departure relative to global trend, and it was estimated that there was a discontinuous zone of local distribution, with areas located in Inner Mongolia, north Xinjiang, and extensive southern provinces experienced a stronger local downward trend. And districts/counties with the local spatiotemporal trend of CVD mortality risk (*exp*(*b*_1*i*_) < 1.0) experienced an approximate or weaker local downward trend compared with national level, and it was mainly located in the majority part of Tibet and central provinces like Hebei, Henan, and Shanxi. Unlike spatial relative risk of CVD mortality at county-level, sex disparities were observed of local spatiotemporal trend, for female population showed more evident local decline trend compared with national trend in male population, especially in northeast provinces like Heilongjiang, Jilin, and Liaoning, as well as western provinces like Tibet (see Additional file 1: Part 4. Fig. S3, Fig. S4).

In Table 1, we further classified the 2844 districts/counties into nine categories, hot spots, cold spots, and warm spots, as well as areas with stronger local trend compared with global trend, weaker local trend compared with global trend, and approximate local trend compared with global trend, based on the two-stage classification rules as previously outlined. Among the 2844 counties of total CVD population, 1144 (40.22%) were classified as hot spots, 1551 (54.54%) as cold spots, and 275 (9.60%) as warm spots. Of the 1144 hot spots, most (492 counties, 43.00%) experienced a stronger local downward trend of CVD mortality risk, while another (453 counties, 39.60%) experienced a weaker local downward trend, and the local trend of 199 counties (17.40%) was approximated to the national downward trend. Additionally, among 1551 cold spots, over a half of counties (801 counties, 51.64%) experienced a stronger local downward and another (459 counties, 29.59%) experienced a weaker local downward trend, and the local trend of 285 counties (18.38%) was approximated to the national downward trend. No obvious sex disparities were observed during the 2-stage classification process (see Additional file 1: Part 4. Table S8).

**Table 1:** Cross classification of spatiotemporal relative risk for CVD mortality (*N*, %)^a^

	Temporal trends			
	Stronger local trend compared with global trend	Weaker local trend compared with global trend	Approximate local trend compared with global trend	Total
**Spatial pattern**				
**Hot spots**	Total: 492 (43.00%)	Total: 453 (39.60%)	Total: 199 (17.40%)	Total: 1144 (100%)
**Cold spots**	Total: 801 (51.64%)	Total: 459 (29.59%)	Total: 285 (18.38%)	Total: 1551 (100%)
**Warm spots**	Total: 135 (49.09%)	Total: 91 (33.09%)	Total: 49 (17.82%)	Total: 275 (100%)

^a^*N* represented to the number of counties that were classified to each of the categories according to the posterior probability estimated by HBSTM during 2-stage classification. The symbol % represented to the percentage of certain number of counties in each of the classification occupied total counties in China

**Fig. 3:**
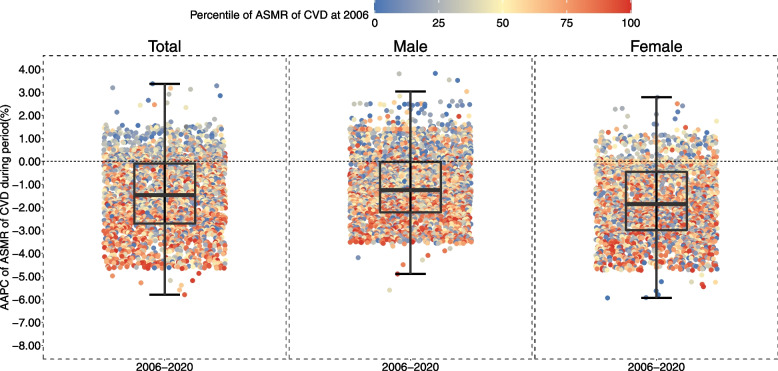
Change in CVD mortality risk at county-level in China, by sex, 2006–2020. Each point showed the median posterior change of CVD mortality in each county in China. Counties were colored by their percentile of ASMR of CVD at the beginning of the study period (e.g., during 2006–2020, they were colored by ASMR of CVD in 2006). The inner box shows the 25th, 50th, and 75th percentiles, and the outer lines the 5th and 95th percentiles

As shown in Fig. 3, the ASMR of CVD decreased during 2006–2020 was smaller in districts/counties where CVD mortality had been comparatively lower in 2006, and vice versa, especially among male population. Specifically, according to the results estimated from Fig. 1, among total population, a gap between lowest and highest county of ASMR of CVD in 2020 (315.62 per 100,000) were smaller than those in 2006 (428.19 per 100,000), while this gap was in male (490.08 per 100,000) and (377.62 per 100,000) in female population. During the study period, CVD decedents in 2539 counties (89.28%) had a mean posterior change in CVD mortality that was negative, while the number was 2472 (86.92%) in men and 2617 (92.08%) in women. However, there were still an around 10% districts/counties were observed to appear an upward trend of CVD mortality risk during the years, and most of them were districts/counties with lowest CVD mortality in 2006 which shaded in blue; also, this feature was more obvious in male than that in female population. As an opposite, counties with a higher CVD mortality were tended to decrease substantially compared with their counterparts, although not all.

### Patterns of nonmedical ecological factors influences on CVD mortality risk

As shown in Table 2, four nonmedical ecological factors, GDP, NTL, NB, and PD at county-level, were incorporated in order to (a) explain the overall spatial pattern of CVD mortality risk and hence to provide evidence as to why some counties had persistent low/high risk and (b) explain the departures of the local trends from the global trend. We have estimated the national and subnational associations of three proxies for eastern, central, and western areas in China separately, and those associations were measured by relative risk (RR). Nationally, NTL was the major influencing factor, a higher GDP and higher NTL at the county-level appeared to be negatively associated with a lower CVD mortality risk (*RR*_*GDP*_=0.92, 95% CI: 0.89, 0.96, *P* < 0.001, with a 10 thousand yuan increase in GDP and the CVD mortality risk will decrease by 8%, *P* < 0.001; and *RR*_*NTL*_ =0.89, 95% CI: 0.83, 0.94, *P* < 0.001, with one unit increase in NTL and the CVD mortality risk will decrease 11%, *P* < 0.001), while NB was estimated not significantly related to CVD mortality. Apart from global level, different patterns of influence were exhibited in the three separate regions of China. Specifically, it was shown that GDP was the significant influencing factor in eastern regions while central and western regions shown an uncertainty relationship between GDP and CVD mortality risk. For NTL, there was a high level of certainty that NTL was negatively associated with CVD mortality risk in subnational levels, for example in eastern areas, the CVD mortality risk decreased by 17% when NTL increased by one unit (*RR*_*NTL*_ =0.83, 95% CI: 0.78, 0.87, *P* < 0.001). Additionally, although NB was estimated to have no significant association with CVD mortality risk at national level, eastern regions shown there were negative association between NB and CVD mortality risk, the CVD mortality decreased by 11% (*RR*_*NB*_=0.89, 95% CI: 0.86, 0.92, *P* < 0.001) when NB increased by one bed per 1000 healthcare institutions, while CVD mortality risk decreased no significantly by 2% (*RR*_*NB*_=0.98, 95% CI: 0.92, 1.04, *P* = 0.319) when NB increased by one unit. PD, however, was estimated to have no statistically significant associations with CVD mortality risk.

**Table 2:** Posterior estimates of associations between nonmedical ecological determinants and CVD mortality risk in China, 2006–2020: estimated from Bayesian multivariable regression model

Region	***RR***_***GDP***_^a^	***RR***_***NTL***_	***RR***_***NB***_	***RR***_***PD***_
**Nationwide**	0.92 (0.89, 0.96) ${P}_{RR_{GDP<1\mid data}}=99.03\%$^bc^	0.89 (0.83, 0.94) ${P}_{RR_{NTL<1\mid data}}=99.99\%$	0.98 (0.94, 1.02) ${P}_{RR_{NB<1\mid data}}=43.09\%$	0.99 (0.95, 1.01) ${P}_{RR_{PD<1\mid data}}=51.22\%$
**Eastern areas**	0.90 (0.88, 0.93) ${P}_{RR_{GDP<1\mid data}}=99.91$%	0.83 (0.78, 0.87) ${P}_{RR_{NTL<1\mid data}}=99.99\%$	0.89 (0.86, 0.92) ${P}_{RR_{NB<1\mid data}}=99.82\%$	0.96 (0.90, 1.02) ${P}_{RR_{PD<1\mid data}}=54.93\%$
**Central areas**	0.99 (0.95, 1.02) ${P}_{RR_{GDP<1\mid data}}=58.94\%$	0.92 (0.85, 1.00) ${P}_{RR_{NTL<1\mid data}}=94.13\%$	0.99 (0.95, 1.04) ${P}_{RR_{NB<1\mid data}}=49.13\%$	0.98 (0.94, 1.02) ${P}_{RR_{PD<1\mid data}}=47.66\%$
**Western areas**	0.96 (0.91, 1.01) ${P}_{RR_{GDP<1\mid data}}=87.51\%$	0.84 (0.75, 0.92) ${P}_{RR_{NTL<1\mid data}}=99.98\%$	0.98 (0.92, 1.04) ${P}_{RR_{NB<1\mid data}}=42.29\%$	0.97 (0.92, 1.01) ${P}_{RR_{PD<1\mid data}}=41.63\%$

^a^The Bayesian model was adjusted for annual average temperature (TEMP, °C), temperature variability (TV, °C), annual average relative humidity (HUMID, %), longitude (LT), altitude (AT), and concentration of PM_2.5_ emission (PM_2.5_, *μg*/*m*^3^), as confounders in main analysis

^b^RRs represented the relative risk of posterior median and its 95% confidence interval (CI), equals to an exponential transformation of the regression coefficients of proxies and CVD mortality

^c^${P}_{RR_{proxies<1\mid data}}$ represented the posterior probability of negative association between different proxies and CVD mortality during 2006 and 2020. In a county in which the entire posterior probability of CVD mortality was negatively associated with those proxies, there was around a 100% posterior probability of a negative association and a 0% posterior probability of a positive association, and vice versa. Meanwhile, posterior probabilities more distant from 50% indicate more certainty

## Discussion

In this population-based study, by using data from NMRIMS in mainland China, for the first time, we used HBSTM to depict spatiotemporal distribution of CVD mortality risk at county-level and quantify its associations between selected nonmedical ecological factors of 2844 Chinese counties from 2006 to 2020. We found that, although China had decreased CVD mortality in recent decade, substantial temporal and spatial heterogeneity still existed, and those inequalities in CVD mortality were narrowed in different regions. The patterns of influences caused by three ecological determinants of the CVD mortality at national and subnational level were identified, including GDP, nighttime light, and number of beds in healthcare institutions.

### Substantial spatiotemporal variations of local CVD mortality distribution at county-level

Little domestic studies at county-level have neither attempted to depict spatiotemporal distribution nor provided evidence to reveal the impacts of nonmedical ecological determinants on CVD mortality [8, 16]. As a further step, this study broadly examined county-level spatiotemporal patterns of CVD mortality and its associated nonmedical ecological factors, and it revealed new patterns not observed when CVD mortality were considered at a larger scale. In addition, our work used the data collected by NMRIMS to identify those characteristics from complicated spatiotemporal coupling process of the CVD mortality. Generally, the Bayesian estimated overall spatial trends for the period 2006–2020 in our study were similar to, though not exactly the same as, the spatial patterns in CVD mortality for China during the same period that were demonstrated in Liu [8] and Wang [16] studies.

A distinct spatial structure with several clusters of CVD mortality and its change over time had been stable during the study period. Three provinces, Tibet, Heilongjiang, and Jilin, showed the highest level of CVD mortality during the study period, while five provinces, Liaoning, Henan, Shaanxi, Hebei, and Xinjiang, demonstrated the second-highest level of CVD mortality. Furthermore, the CVD mortality in most of northeast areas, north to central areas, and western China was at a higher level than the national average, with the hotspots classified by the posterior probability of the spatial relative risk being greater than 1.0. Despite the generally downward trend of CVD mortality, it nonetheless presented a highly spatial heterogeneity. Different local trends in county-level CVD mortality appeared a decentralized distribution throughout China. In previous study, Wang [16] have calculated the annual rate of change in premature mortality burden of CVD mortality at provincial level from 2005 to 2020. However, the spatial patterns of local trends estimated by our study this time, decomposed from the overall trend and considered local spatiotemporal correlation, possessed a more striking spatial structure than those concluded by Wang [16] study, which were calculated independently for each province. We argued that the local trends with distinct spatial structure may have a closer relation to reality. Specifically, this study noticed that those counties with larger downward change tended to be places where CVD mortality was already relatively higher at the beginning of study period compared with their counterparts. Nevertheless, some counties dispersedly located in southern and northwest exhibited a comparatively stronger downward trend than the national average, whereas some other counties dispersedly located in underdeveloped areas from western and southwestern regions, like Tibet, Qinghai, Gansu, Shanxi, and Shaanxi, not only shown a higher CVD mortality at the beginning of study period but also presented a weaker decrease compared with national average. Herein, for shifts and spatial variations at such small scale, it might be largely due to unequally distributed of risk factors of CVD mortality and healthcare services [4, 20, 26].

### Significant associations between nonmedical ecological factors and CVD mortality

Our study shed light on the associations between nonmedical ecological factors and CVD mortality. The results suggested that, nationally, GDP and NTL were major influencing factors among selected proxies. On the basis of magnitude of associations between those proxies and CVD mortality, the effects of NTL on CVD mortality were relatively higher than GDP. Differences of effects among GDP and NTL might be related to the reasons that NTL was an integrated indicator which could be able to comprehensively reflected the level of regional socioeconomic development, and previous studies have also proved that districts with satisfied local socioeconomic conditions tended to present better population health status and lower CVD mortality. At subregional level, a nearly absolute negative association between proxies and CVD mortality was observed particularly in eastern areas for both GDP and NTL, while in central areas, less certainties of negative association were generated, which we speculated the effects might be related to the joint impacts by other proxies, such as lifestyle and environmental factors [25, 26, 28, 48–51]. As for NB, although it was not the principal factor at national level, it was estimated as major influencing factors in eastern areas instead, and it was rationality to understand a high quality medical and healthcare capacity, which was expressed by NB, would largely improve the effects of treatment and prognosis among CVD patients. Yet, the relationship was not significantly estimated at central and western areas. We speculated that it might be related to disadvantaged in medical technologies for inpatient severe CVD cases and/or insufficient allocation of pre-hospital emergency resources among acute CVD patients, and those associations between NB and CVD mortality among those areas might be shadowed by unequal distribution by other potential factors [4, 5, 26, 27, 48, 49]. However, we did not observe a statistically significantly association between PD and CVD mortality, for which it might relate to the data quality or the disease status might not influenced by population distribution or characteristics in a specific area. And little literatures reported the same findings.

Apart from aforementioned nonmedical ecological factors, the magnitude and trends of CVD epidemic variations in China were largely affected by a number of factors for areas. These factors can best be understood in three major categories [9]: (1) variations in the level of exposure to metabolic, behavioral, and environmental risks for the population; (2) variations in the delivery of interventions that can modify risks due to these exposures; and (3) delivery of high-quality emergency services and acute medical care that improve health outcomes when CVD events occur. To be more specific, CVD risk factors still remained prevalent among community settings, such as smoking, drinking, physical inactivity, and elevated blood pressure, especially under the era of population aging [8]. While in clinical settings, availability and affordability of medications, the quality of medical care, the adherence to treatment among patients, and the proper handling for secondary prevention after discharge provided by professionals might all exert influences on the issue [25, 26, 48]. Further investigation is needed to better understand regional variations in the factors that lead to CVD mortality at county level in China.

### Implications

Current spatiotemporal characteristics of Chinese county-level CVD mortality differences might be an interplay between multiple factors, which are deeply rooted in large-scale socioeconomic and lifestyle changes in the whole society. As the most populous country worldwide facing substantial unequally distribution in population aging, lifestyle, and socioeconomic development, we supposed that CVD mortality might sustain its diversities in spatial patterns and gradually downward trend in the near future [16]. A broader exploration of the quality of both facility-based and community-based health interventions in the highest-risk CVD mortality counties would be an important step in reducing CVD mortality differences in China [48]. Fortunately, counties with successful implementation of interventions have been well documented nationwide, such as the establishment and enforcement of National Demonstration Areas for Comprehensive Prevention and Control of Chronic Diseases. On the other hand, to reduce population mortality and improve health equity has long been a government priority, and Healthy China 2030 included justice and equity as one of the four core principle; thus, the rationality of healthcare resource allocation is urgently need to address substantial health inequity [48, 50].

### Strengths and limitations

A main strength of our study was that, it was known as the first presentation of small scale research for CVD mortality across China in 2844 counties over an extended period between 2006 and 2020 when substantial change in economic, health, and society occurred in the country. Since the data consisted of aggregated counts of outcomes and covariates, typically, disease mapping and/or ecological regression can be specified; it could be generated in the state-of-the-art Bayesian framework by extending the concept of hierarchical structure, allowing to account for similarities based on the neighborhood or on the distance at area-level. In summary, there are several advantages by using Bayesian approach in current study: first, the specification of prior distributions allows the formal inclusion of information that can be obtained through previous studies or from expert opinions; thus, posterior probability that a parameter does/does not exceed a certain threshold is easily obtained from posterior distribution, providing a more intuitive and interpretable quantity instead of a frequentist *P*-value. Second, it is easy to specify a hierarchical structure on the data and/or parameters within Bayesian approach, which presents the added benefits of making predictions for new observations and missing data imputation relatively straightforward. Third, in this spatiotemporal analysis, Bayesian model can disassemble the overall spatial relative risk, overall temporal trend, and local trend from the complex space-time coupling process to closely investigate the spatiotemporal heterogeneities in CVD mortality at county-level, in order to obtain robust and comparable yearly estimates together with uncertainties for small areas [15, 34, 36–41].

However, this study was also subjected to several limitations. As for study design, it was important to notice that ecological fallacy may exist when analyzing county-level nonmedical determinants of CVD mortality since it revealed how drivers of disparate CVD mortality operate at local and neighborhood level but did not establish robust evidence for inferring causation [19]. As for mortality and population data, first, both of them were subjected to numerator-denominator bias, which referred to deaths occurred and individuals lived within a small-area may be missed or allocated to the wrong districts/counties. For example, vital registration attributed deaths to county of residence at the time of death occurred, and no correction to migration between counties, while population data did not consider internal migration either and did not necessarily obtain the health of population living in an area for their entire lifetime [19]. Second, ascertainment bias caused by reporting accuracy of COD may attenuate the quantity and quality of CVD mortality estimation [19]. Third, under-reporting of CVD mortality still existed although we conducted under-reporting adjustment, and it might also increase the uncertainties or even related to underestimation of spatially CVD mortality disparities [16, 19]. As for nonmedical ecological determinants, first, it may not fully capture the comprehensive interplay of nonmedical determinants process towards CVD mortality disparities; hence, omitted variables and potential confounders were inevitable. Indeed, apart from factors like GDP, NTL, NB, and PD, there were certainly other influencing factors that should form the focus of future research and measure detailed conditions, such as accessibility of healthcare services and treatments implementation at county-level [48, 50]. Second, inadequate acquisition of long-term cumulative effects of those indicators may left some attributions unexplained. Third, data quality of publicly available indicators might lead to uncertainties, and the approaches we used to perform multiple imputation might also incompletely reflect the actual situation [19]. As for results reporting, we failed to report the CVD mortality by its subcategories, mainly due to the target was to demonstrate the total CVD, and it was regarded as the pilot study of series of upcoming small scale epidemiological studies nationwide. As for methods, first, although indicators screening was relied on literature findings, the mechanism of selected ones might spatially interact with each other through mediating and/or moderating effects actually; thus, the linear-based modeling approach may insufficiently demonstrate the association between nonmedical ecological determinants and CVD mortality disparities [19]. Second, the random walk priors and random effects distribution under Bayesian framework that removed variability due to rare events by sharing information across spatial units could attenuate true variations, and the true extents of CVD mortality inequities across counties was likely to be larger than estimated in the analysis [15].

In response to those shortcomings, considerations should be raised to improve and consolidate a sophisticated theoretical framework of CVD mortality associated nonmedical ecological determinants with an upstream influencing mechanism in the future. In additional to these, expanding the data accessibility and availability should be enhanced, such as increase the data quality of mortality data collected by NMSS, and methodology for dealing with missing data and non-linear modeling process should also be improved to understand the results more precisely with qualified robustness [19].

## Conclusions

In this analysis of CVD mortality risk of 2844 counties in mainland China from 2006 through 2020, substantial between-county discrepancies of CVD mortality distribution were observed. Nonmedical ecological determinants were estimated to significantly explain the overall and local spatiotemporal patterns of this CVD mortality risk. Targeted considerations are needed to integrate primary care with clinical care through intensifying further strategies to narrow unequally distribution of CVD mortality at local scale. The approach to county-level analysis with small-area models used in this study has the potential to provide novel insights into China disease-specific mortality spatiotemporal trends and their differences across geographic regions.

## Supplementary Information

**Additional file 1: Table S1.** List of data source and curation of mortality and population data. **Table S2.** 2010 Chinese census population. **Table S3.** List of data source, calculation and curation of nonmedical ecological determinants. **Table S4.** Cross classification for CVD mortality risk identification. **Table S5.** Included number of districts/counties and CVD death counts nationwide, 2006-2020. **Table S6.** Model performance and selection. **Table S7.** Posterior distribution of parameters estimated by HBSTM of CVD mortality at county-level in China, 2006-2020. **Table S8.** Cross classification of spatiotemporal relative risk for CVD mortality, by sex (N, %). **Fig. S1.** ASMR of CVD and its change at county-level in China, male, 2006-2020. **Fig. S2.** ASMR of CVD and its change at county-level in China, female, 2006-2020. **Fig. S3.** Spatiotemporal patterns of relative risk of CVD mortality at county-level in China, male, 2006-2020. **Fig. S4.** Spatiotemporal patterns of relative risk of CVD mortality at county-level in China, female, 2006-2020.

## Data Availability

For raw data extracted from National Mortality Surveillance System and under-reporting surveys used in current study, they were not publicly available due to data sharing regulations established by China CDC, but they were available from the corresponding authors on reasonable request. For raw data extracted from multi-source of nonmedical ecological determinants, they were publicly available through National Bureau of Statistics, national or regional statistics yearbook, and specific literatures. Detailed information of data sharing and accessibility was reported in the additional file. In reference to programs both for analysis and derivation of the data, they were not publicly available due to intellectual property for statistical analysis regulations established by China CDC, but they were available from the corresponding authors on reasonable request.

## Abbreviations

CVD: Cardiovascular disease
COD: Cause of death
NMRIMS: National Mortality Registration Information Management System
CDC: Center for Disease Control and Prevention
NMSS: National Mortality Surveillance System
PLADs: Provincial-level administrative divisions
GDP: Gross domestic product
NTL: Nighttime light
NB: Number of beds in health care institutions
PD: Population density
TEMP: Annual average temperature
TV: Temperature variability
HUMID: Average relative humidity
LT: Longitude
AT: Altitude
PM2.5: Concentration of PM_2.5_ emission
URR: Under-reporting rate
ICD-10: International Classification of Disease 10th Edition
HBSTM: Hierarchical Bayesian spatiotemporal model
SMR: Standardized mortality ratio
CAR: Conditional autoregressive
ASMR: Age-standardized mortality rate
AAPC: Average annual percent change
