# Supplementary material for: Spatiotemporal trends and ecological determinants of cardiovascular mortality among 2844 counties in mainland China, 2006–2020: a Bayesian modeling study of national mortality registries
Source: BMC Med. 2022 Nov 30;20:467. doi: 10.1186/s12916-022-02613-9 (PMC9714200; doi:10.1186/s12916-022-02613-9)
Supplement: Supplementary file 1 — Additional file 1: Table S1. List of data source and curation of mortality and population data. Table S2. 2010 Chinese census population. Table S3. List of data source, calculation and curation of nonmedical ecological determinants. Table S4. Cross classification for CVD mortality risk identification. Table S5. Included number of districts/counties and CVD death counts nationwide, 2006-2020. Table S6. Model performance and selection. Table S7. Posterior distribution of parameters estimated by HBSTM of CVD mortality at county-level in China, 2006-2020. Table S8. Cross classification of spatiotemporal relative risk for CVD mortality, by sex (N, %). Fig. S1. ASMR of CVD and its change at county-level in China, male, 2006-2020. Fig. S2. ASMR of CVD and its change at county-level in China, female, 2006-2020. Fig. S3. Spatiotemporal patterns of relative risk of CVD mortality at county-level in China, male, 2006-2020. Fig. S4. Spatiotemporal patterns of relative risk of CVD mortality at county-level in China, female, 2006-2020. [file 12916_2022_2613_MOESM1_ESM.docx]

# Additional file 1

Additional file 1 appendix to:

Spatiotemporal trends and ecological determinants of cardiovascular mortality among 2844 counties in mainland China, 2006-2020: A Bayesian modelling study of national mortality registries

## Contents

Part 1. Description of data source

Part 2. Data quality control

Part 3. Methods of hierarchical Bayesian spatiotemporal model

Part 4. Additional analysis and results

## List of tables and figures

Table S1. List of data source and curation of mortality and population data

Table S2. 2010 Chinese census population

Table S3. List of data source, calculation and curation of nonmedical ecological determinants

Table S4. Cross classification for CVD mortality risk identification

Table S5. Included number of districts/counties and CVD death counts nationwide, 2006-2020

Table S6. Model performance and selection

Table S7. Posterior distribution of parameters estimated by HBSTM of CVD mortality at county-level in China, 2006-2020

Table S8. Cross classification of spatiotemporal relative risk for CVD mortality, by sex (N, %)

Figure S1. ASMR of CVD and its change at county-level in China, male, 2006-2020

Figure S2. ASMR of CVD and its change at county-level in China, female, 2006-2020

Figure S3. Spatiotemporal patterns of relative risk of CVD mortality at county-level in China, male, 2006-2020

Figure S4. Spatiotemporal patterns of relative risk of CVD mortality at county-level in China, female, 2006-2020

## Part 1. Description of data source

### 1. Disease Surveillance Point system

The Disease Surveillance Point system (DSPs) in China was piloted in 1978 with 2 sites in Beijing and fully established in 1989 covering initially 71 sites involving 29 provinces. In 1990, Chinese Academy of Preventive Medicine (currently known as Chinese Center for Disease Control and Prevention, China CDC) re-selected the points in DSPs and it was enlarged to 145 sites covering 10 million population (accounted for 1% of total Chinese population) under the multi-stage stratified cluster random sampling framework, thereafter, the DSPs routinely collected registries of vital, population and mortality statistics. In 2004, in order to accommodate the societal and economic development during this period, the DSPs was further adjusted to 161 sites covering 73 million population (accounted for 6% of total Chinese population). For each of the adjustment during the years, the population characteristics of the selected surveillance points were compared to the census population to ensure its nationally representativeness. The sampling strategy and the characteristics of DSPs have been described in detail elsewhere along with the quality control measures and the procedures for collecting data, coding the cause of death (COD) and determining the underlying COD.

### 2. Vital Registration system

The Vital Registration system (VRs) in China was established in the 1950s to collect mortality data in 13 cities and hosted by the Ministry of Health (currently known as National Health Commission, NHC). In 2000, the population under surveillance was around 110 million and the VRs covered 15 large cities, 21 medium-sized or small cities and 90 counties drawn from 15 provinces and municipalities. In 2012, the VRs had expanded to include 319 sites (138 counties and 181 districts) in 22 provinces covering about 230 million people, mostly in eastern and central areas of the country, among which, 42 counties in the VRs overlapped with population catchments in the DSPs.

### 3. National Mortality Surveillance System

In 2013, National Health and Family Planning Commission (currently known as NHC) combined the DSPs in China CDC and Vital Registration (VR) system hosted by the Ministry of Health (currently known as NHC) to create an integrated National Mortality Surveillance System (NMSS). After which, NMSS covered 324 million population (24% of the total population of the country) from 605 sites in 31 provinces. The goals were to integrate and rationalize the health resources expended on these systems and to accelerate the development of a complete vital registration and mortality surveillance system covering the entire population of China. Detailed descriptions of stratification, representativeness determination, surveillance points death information collection and certificate, coding and determining underlying COD, as well as procedures of quality control, have been reported elsewhere. From 2006 to 2020, the NMSS have collected over 8 million death cases nationwide in total.

### 4. National Mortality Registration Information Management System

Other than surveillance points included in NMSS, those rest of counties/districts in China that neither belongs to VRs nor DSPs have also reported death cases directly through National Mortality Registration Information Management System (NMRIMS), thus NMRIMS have collected death records from nearly all counties/districts across the country. From 2006 to 2020, the NMRIMS have collected over 72 million death cases nationwide in total.

### 5. Data source and curation of mortality and population data

#### Table S1. List of data source and curation of mortality and population data

| **Type** | **Data source** | **Missing data** | **Imputation approach** |
| --- | --- | --- | --- |
| Morality data | NMSS, NMRIMS  Year: 2006-2020.  Coverage: All districts/counties that reported death cases in mainland China.  Availability: Housed in China CDC and was only availability under reasonable requests. | No missing data. | NA. |
| Under-reporting data | Under-reporting field surveys for NMSS  Year: 2006-2017.  Coverage: All surveillance points within NMSS in mainland China.  Availability: Housed in China CDC and was only availability under reasonable requests. | Year: 2018-2020.  Coverage: All surveillance points within NMSS in mainland China. | Splines regression. |
| Population data | National Data from National Bureau of Statistics  Year: 2006-2020.  Coverage: All districts/counties in mainland China.  Availability: Housed in China CDC and was only availability under reasonable requests. | No missing data. | NA. |
| Population data for rate standardization | 2010 Chinese census  Year: 2010  Coverage: population census  Availability: Publicly available in National statistics yearbook. | No missing data. | NA. |

#### Table S2. 2010 Chinese census population

| **Age groups** | **Population (person)** |
| --- | --- |
| 0- | 13814161 |
| 1-4 | 61859647 |
| 5-9 | 70965292 |
| 10-14 | 74886034 |
| 15-19 | 99832077 |
| 20-24 | 127462868 |
| 25-29 | 101227255 |
| 30-34 | 97423899 |
| 35-39 | 118332523 |
| 40-44 | 124996066 |
| 45-49 | 105811899 |
| 50-54 | 78936414 |
| 55-59 | 81509713 |
| 60-64 | 58820012 |
| 65-69 | 41213084 |
| 70-74 | 33052450 |
| 75-79 | 23901354 |
| 80-84 | 13399403 |
| 85 and above | 7627526 |
| Total population | 1335071677 |

### 6. Data source, calculation and curation of nonmedical ecological determinants

#### Table S3. List of data source, calculation and curation of nonmedical ecological determinants

| **Nonmedical ecological determinants** | **Calculation methods** | **Data curation** | | |
| --- | --- | --- | --- | --- |
|  |  | **Data source** | **Missing data** | **Imputation approach** |
| NPP-VIIRS-like nighttime light data (NTL) | It is radiational signal in the nighttime to detect artificial electric light equipped in most buildings and infrastructures by remote sensors. | A composition source of Defense Meteorological Satellite Program Operational Linescan System (DMSP-OLS) stable nighttime light data and Suomi National Polar-orbiting Partnership-Visible Infrared Imaging Radiometer Suite (NPP-VIIRS) nighttime light data.  Year: 2006-2018. | Year: 2019, 2020.  Areas: Some of the districts/counties in mainland China. | Multiple imputation (MI) with predictive mean matching (PMM)  approach. |
| Per capita gross domestic product (GDP, 10 000 yuan per person) | Equals to the ratio of the absolute value of GDP to the average population in that year. | County-level statistics yearbook.  Year: 2006-2020. | No missing data. | NA. |
| Number of beds in health care institutions per 10 000 persons (NB, units) | Equals to the number of beds of medical and healthcare institutions divided by population. | County-level statistics yearbook.  Year: 2006-2018. | Year: 2019, 2020.  Areas: Some of the districts/counties in mainland China. | MI with PMM approach. |
| Population density (PD, person per 1 square kilometer, person) | Equals to the number of people per unit of land area. | County-level statistics yearbook.  Year: 2006-2020. | Year: 2020.  Areas: Some of the districts/counties in mainland China. | MI with PMM approach. |
| Annual average temperature (TEMP, ℃) | Equals to add up the daily mean temperatures in the year and dividing by number of days in the year. | China Meteorological Data Sharing Service System.  Year: 2006-2019. | No missing data. | NA. |
| Temperature variability (TV, ℃) | Equals to the standard deviation of daily average temperatures. | China Meteorological Data Sharing Service System.  Year: 2006-2020. | No missing data. | NA. |
| Annual average relative humidity (HUMID, %) | Equals to the average of daily relative humidity of a year. | China Meteorological Data Sharing Service System.  Year: 2006-2020. | No missing data. | NA. |
| Longitude (LT) | NA. | National Platform for Common Geospatial Information Services; National Geomatics Center of China  Year: NA. | No missing data. | NA. |
| Altitude (AT) | NA. | National Platform for Common Geospatial Information Services; National Geomatics Center of China  Year: NA. | No missing data. | NA. |
| Concentration of PM_2.5_ (PM_2.5,_ $\mu g/m^{3}$) | NA. | NASA Global Modeling and Assimilation Office (GMAO), Goddard Earth Sciences Data and Information Services Center (GES DISC). | No missing data. | NA. |

## Part 2. Data quality control

In the consideration of the issues of newly-added, cancellation and alternation of the administrative codes in each of the district/county, before data quality control, we thus unified and included district/counties throughout 2006-2020 to acquire consistent and identical information during the years.

For the first step, we have reviewed, compared and evaluated the data quality of each county to exclude certain ones that were considered to be seriously under-reported and might affect overall results. We made the under-reporting-adjusted all-cause mortality rate of lower than 4.5‰ as exclusion criteria for those counties belongs to VRs and DSPs earlier than 2012. Since 2013, we made the criteria of 5‰. For counties that neither belongs to VRs nor DSPs, we made the criteria of 3‰ during 2006-2020.

For the second step, we constructed a linear regression to fit the association and quantify the magnitude between under-reporting-adjusted all-cause mortality rate/life expectancy at 605 surveillance points in NMSS and per capita gross domestic product (GDP) during 2013-2020 at both county and provincial level. Afterwards, we evaluated all districts/counties across China during 2006-2020 through applying the equation estimated previously to acquire expected value of those included areas which meet the criterion of quality control. Among which, for the associations established within 605 surveillance points, we used the results estimated by linear regression performed at district/county level directly; for the associations established without 605 surveillance points, we used the results estimated by linear regression performed at provincial level. Finally, we included districts/counties with a range of $\pm$10% difference between observed value and expected value of all-cause mortality rate and life expectancy in the main analysis of this study.

## Part 3. Methods of hierarchical Bayesian spatiotemporal model

### 1. Model priors and hyperpriors

During Bayesian modelling process, prior and hyperprior were assigned for all parameters main model. For prior distribution specifications, we assumed that the overall CVD mortality risk $\alpha$ and overall rate of CVD mortality risk change $b_{0}$ followed a uniform distribution. As for overall spatial random effect $s_{i}$ and local departure from global trend $b_{1i}$, we assigned a conditional autoregressive (CAR) prior with a spatial weight matrix $W$ to impose spatial structure. Specifically, $W$ is a matrix of size $N\times N$, where its diagonal entries $w_{ii}=0$ and the off-diagonal entries $w_{ij}=1$ if county $i$ and $j$ share a common boundary and $w_{ij}=1$otherwise. Herein, CAR prior on the spatial random effect implies that the adjacent counties tended to have changes in CVD mortality risk that were more alike than is the case for counties that were far apart. Besides, both of the nonlinear temporal trends $v_{t}$ and overdispersion parameter $\varepsilon_{it}$followed a normal distribution. As suggested by Gelman, for hyperprior distribution specifications, we assigned a strictly positive half Gaussian prior to all random effect standard deviations, and precision parameter followed Gamma noninformative distribution. Details of prior and hyperprior distribution parameter were specified as follows:

| **Distribution of priors** |
| --- |
| $\alpha\sim dflat(),b_{0}\sim dflat(),s_{i}\sim CAR\left( \sigma_{s}^{2} \right), u_{i}\sim N\left( 0,\sigma_{u}^{2} \right),$ |
| ${b_{1i}\sim CAR\left( \sigma_{b_{1i}}^{2} \right),v}_{t}\sim N\left( 0,\sigma_{v}^{2} \right),\varepsilon_{it}\sim N\left( 0,\sigma_{\varepsilon}^{2} \right)$ |
|  |
| **Distribution of hyperpriors and precision parameters** |
| $\sigma_{s}\sim U\left( 0,100 \right),{\sigma_{u}\sim U\left( 0,100 \right),\sigma}_{b_{1}}\sim U\left( 0,100 \right),\sigma_{v}\sim U\left( 0,100 \right),\sigma_{\varepsilon}\sim U\left( 0,100 \right)$ |
| $\frac{1}{\sigma_{s}^{2}}\sim Gamma\left( 0.5,0.0005 \right),\frac{1}{\sigma_{b_{1}}^{2}}\sim Gamma\left( 0.5,0.0005 \right)$ |
| $\frac{1}{\sigma_{v}^{2}}\sim Gamma\left( 0.5,0.0005 \right),\frac{1}{\sigma_{\varepsilon}^{2}}\sim Gamma\left( 0.5,0.0005 \right)$ |

### 2. Model selection

We used deviance information criterion (DIC) to evaluate the goodness of fit of the models. DIC is a Bayesian-based information criterion, which is regarded as the generalization hierarchy model of Akaike information criterion (AIC) and Bayesian information criterion (BIC). DIC is interpret as follows:

| $D(\theta)=-2logf(Y\vert\theta)+2logh(Y)$ | (1) |
| --- | --- |
| $\bar{D}=E_{\theta\vert Y}[D]$ | (2) |
| $pD=E_{\theta\vert Y}[D]-D(E_{\theta\vert Y}[D])= \bar{D}-D(\bar{\theta})$ | (3) |
| $DIC=pD+\bar{D}$ | (4) |

In Equation (1) to (4), $Y$ denotes the observed data; $\theta$ denotes the unknown parameters of model; $f(Y|\theta)$ denotes the likelihood function; $h(Y)$ denotes the constant; $\bar{D}$ denotes the indicator to quantify the model performance between observed data and expected data, the lower the $\bar{D}$ is, the better the model constructed; $pD$denotes the effective number of parameters which is used to assess the complexity of the models, the higher the $pD$ is, the better the model performance is.

### 3. CVD mortality risk classification

We used posterior probabilities estimated by HBSTM to classify CVD mortality risk at county-level. Among which, $(exp(s_{i}+u_{i}))$ denotes the logarithm of CVD death counts over time in county $i$ relative to national average, $data$ denotes the observed data, $p(exp(s_{i}+u_{i})>1|data)$ denotes the posterior probability of relative risk of CVD mortality larger than 1. $p(b_{1i}>0|h_{i},data)$ denotes the posterior probability of local trend of CVD mortality risk in county $i$ relative to national average larger than 0. Therefore, we demonstrated local CVD mortality risk by using the $p(exp(s_{i}+u_{i})>1|data)$ and $p(b_{1i}>0|h_{i},data)$. Specifically, at the first stage, we defined a county as a hot spot ($h_{1})$ if $p(exp(s_{i}+u_{i})>1|data)$was greater than 0.8 and as a cold spot ($h_{2}$) if $p(exp(s_{i}+u_{i})>1|data)$was less than 0.2, besides, we defined the other counties as warm spots ($h_{3}$). At the second stage, we further classified a county under each risk category in the first stage into one of the three trend patterns by using estimates of local slopes $b_{1i}$, namely, a county with a faster increasing local trend than global trend if $p(b_{1i}>0|h_{i},data) >0.8$, a decreasing trend relative to the global trend if $p\left( b_{1i}>0 | h_{i},data \right)<0.2$, and a local trend not differing from the global trend if 0.2 < $p\left( b_{1i}>0 | h_{i},data \right)$ < 0.8. The cross classification for CVD mortality risk identification was shown in Table S4.

#### Table S4. Cross classification for CVD mortality risk identification

| **Temporal trend**  **Spatial pattern** | **Stronger local trend compared with global trend** | **Weaker local trend compared with global trend** | **Approximate local trend compared with global trend** |
| --- | --- | --- | --- |
| **Hot spots** | $p\left( exp\left( s_{i}+u_{i} \right)>1 \vert data \right)>0.8$ and  $p(b_{1i}>0\vert h_{i},data) >0.8$ | $p\left( exp\left( s_{i}+u_{i} \right)>1 \vert data \right)>0.8$ and $p(b_{1i}>0\vert h_{i},data) <0.2$ | $p\left( exp\left( s_{i}+u_{i} \right)>1 \vert data \right)>0.8$ and  $0.2<p(b_{1i}>0\vert h_{i},data)<0.8$ |
| **Cold spots** | $p\left( exp\left( s_{i}+u_{i} \right)>1 \vert data \right)<0.2$ and  $p(b_{1i}>0\vert h_{i},data) >0.8$ | $p\left( exp\left( s_{i}+u_{i} \right)>1 \vert data \right)<0.2$ and  $p(b_{1i}>0\vert h_{i},data) <0.2$ | $p\left( exp\left( s_{i}+u_{i} \right)>1 \vert data \right)<0.2$ and  $0.2<p(b_{1i}>0\vert h_{i},data)<0.8$ |
| **Warm spots** | $0.2<p\left( exp\left( s_{i}+u_{i} \right)>1 \vert data \right)<0.8$ and $p(b_{1i}>0\vert h_{i},data)>0.8$ | $0.2<p\left( exp\left( s_{i}+u_{i} \right)>1 \vert data \right)<0.8$ and $p(b_{1i}>0\vert h_{i},data) <0.2$ | $0.2<p\left( exp\left( s_{i}+u_{i} \right)>1 \vert data \right)<0.8$ and $0.2<p(b_{1i}>0\vert h_{i},data)<0.8$ |

## Part 4. Additional analysis and results

### 1. Characteristics of number of districts/counties and CVD death counts

#### Table S5. Included number of districts/counties and CVD death counts nationwide, 2006-2020

| **Year** | **Number of total districts/counties** | **Number of districts/counties with consistent administrative code** | **Number of total CVD death counts** | **Number of included districts/counties** | **Number of included CVD death counts** |
| --- | --- | --- | --- | --- | --- |
| 2006 | 2698 | 2656 | 319393 | 2108 | 266331 |
| 2007 | 2726 | 2656 | 536655 | 2108 | 449125 |
| 2008 | 2762 | 2656 | 871995 | 2108 | 736514 |
| 2009 | 2767 | 2656 | 966413 | 2108 | 816951 |
| 2010 | 2779 | 2656 | 1233730 | 2108 | 1048059 |
| 2011 | 3003 | 2656 | 1457280 | 2108 | 1240635 |
| 2012 | 3037 | 2656 | 1759456 | 2108 | 1507723 |
| 2013 | 3065 | 2656 | 2140217 | 2108 | 1832262 |
| 2014 | 3039 | 2656 | 2419714 | 2108 | 2072438 |
| 2015 | 3014 | 2656 | 2666816 | 2108 | 2288064 |
| 2016 | 2977 | 2656 | 2981827 | 2108 | 2564282 |
| 2017 | 2955 | 2656 | 3245246 | 2108 | 2791597 |
| 2018 | 2929 | 2656 | 3495099 | 2108 | 2994609 |
| 2019 | 2772 | 2656 | 3683660 | 2108 | 3158975 |
| 2020 | 2720 | 2656 | 3891973 | 2108 | 3333311 |

### 2. Model performance, selection and general parameter estimation

In this study, on the basis of CVD mortality for total population at county level in China during 2006 to 2020, Table S6 represented the comparisons of model performance provided with different model selections. Among which, Model 1 was specified as only spatial model with spatial terms; Model 2 was specified as spatial and temporal model which comprised both spatial and temporal terms; Model 3 was specified as spatiotemporal model with spatial, temporal and spatiotemporal interactions terms. As for model performance, Model 1 was the simplest model since it had shown the smallest effective number of parameters. Although Model 3 shown a higher value of effective number of parameters, the it shown the highest goodness of fit among all models because of its relative lower DIC and $\bar{D}$. Besides, it comprised all possible terms to facilitate the variations of spatiotemporal process. Therefore, Model 3 was selected as the preferred model in the main analysis.

#### Table S6. Model performance and selection

| **Model** | **Model type** | **Model specification** | **DIC ^*^** | $\bar{\boldsymbol{D}}$ **^*^** | $\boldsymbol{pD}$ **^*^** |
| --- | --- | --- | --- | --- | --- |
| Model 1 | Spatial model | $log\left( \theta_{it} \right)=\alpha+s_{i}+u_{i}$ | 33478.77 | 31715.84 | 17790.09 |
| Model 2 | Spatial + temporal model | $log\left( \theta_{it} \right)=\alpha+s_{i}+u_{i}+b_{0}t^{*}+v_{t}$ | 2113.39 | 19319.89 | 19487.27 |
| Model 3 | spatial + temporal + spatiotemporal interaction model | $log\left( \theta_{it} \right)=\alpha+s_{i}+u_{i}+b_{0}t^{*}+v_{t}+b_{1i}t^{*}+\varepsilon_{it}$ | 7769.19 | 6499.49 | 21521.13 |

**Footnotes:**

^*^ DIC represents deviation information criteria which is used to evaluate the goodness of fit of the models. $\bar{D}$ represents the average mean of posterior probability which is used to fit the spatiotemporal models. $pD$ represents the effective number of parameters which is used to assess the complexity of the models.

Meanwhile, the posterior distribution of parameters estimated by HBSTM of mortality at county-level in China during 2006-2020 was shown in Table S7.

#### Table S7. Posterior distribution of parameters estimated by HBSTM of CVD mortality at county-level in China, 2006-2020

| **Parameters ^*^** | **Mean (SD)** | **95% CI** |
| --- | --- | --- |
| $\alpha$ | 1.20 (0.02) | 1.17, 1.23 |
| $b_{0}$ | 159.89 (7.28) | 145.42, 175.21 |
| $\sigma_{s}^{2}$ | 7.96 (0.38) | 7.25, 8.74 |
| $\sigma_{u}^{2}$ | 10.60 (1.35) | 8.28, 13.60 |
| $\sigma_{v}^{2}$ | 69.73 (34.30) | 26.31, 157.27 |
| $\sigma_{b_{1i}}^{2}$ | 6.61 (0.24) | 6.15, 7.09 |
| $\sigma_{\varepsilon}^{2}$ | 169.17 (8.14) | 153.34, 187.68 |

**Footnotes:**

^*^ SD: standard deviation; CI: confidence interval; $\alpha$ represented the overall CVD mortality risk; $b_{0}$ represented the global linear temporal trend of CVD mortality; $\sigma_{u}^{2}$ represented the posterior distribution of spatially unstructured random effects; $\sigma_{v}^{2}$ represented the posterior distribution of local nonlinear trend; $\sigma_{b_{1i}}^{2}$ represented the posterior distribution of local departure from global trend; $\sigma_{\varepsilon}^{2}$ represented the posterior distribution of spatiotemporal stochastic noise.

###
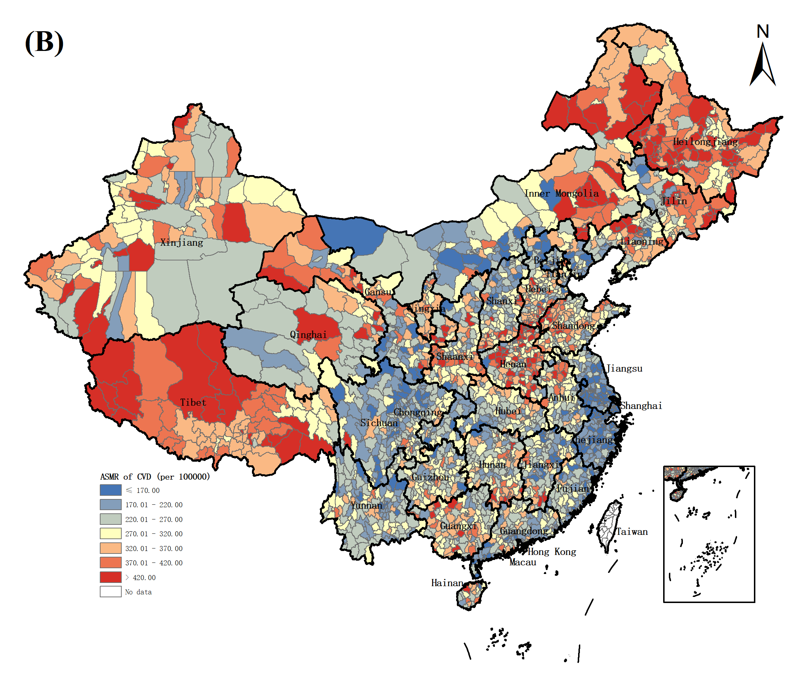

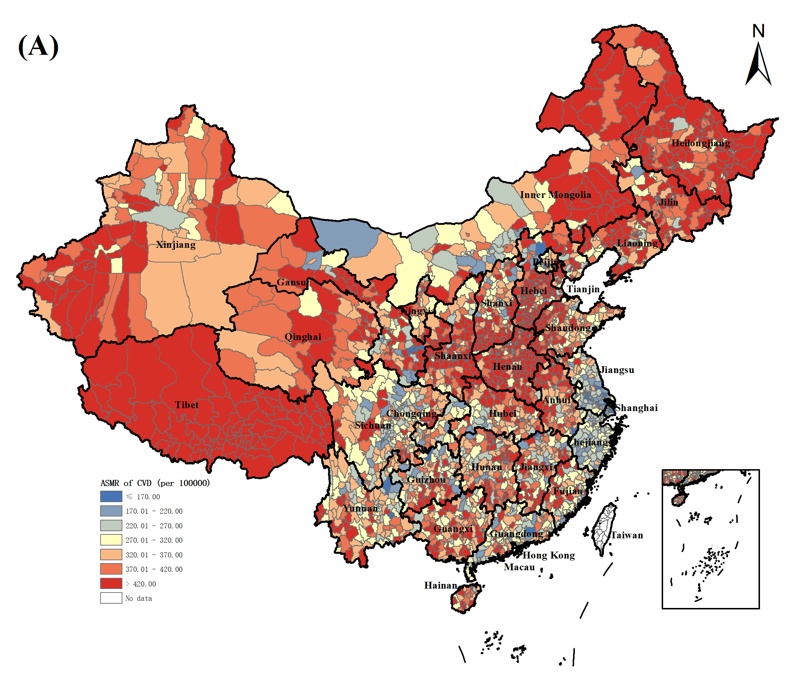
3. ASMR of CVD and its change at county-level in China, by sex, 2006-2020


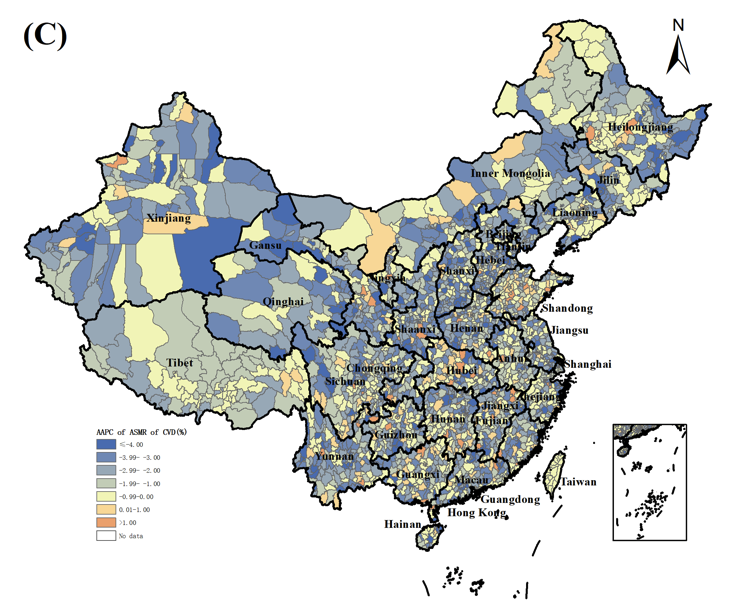


#### Figure S1. ASMR of CVD and its change at county-level in China, male, 2006-2020

(A) ASMR of CVD in 2006, per 100000

(B) ASMR of CVD in 2020, per 100000

(C) AAPC of ASMR of CVD during 2006-2020 (%)

**Footnotes:** ASMR: age-standardized mortality rate; CVD: cardiovascular disease; AAPC: annual average percent change


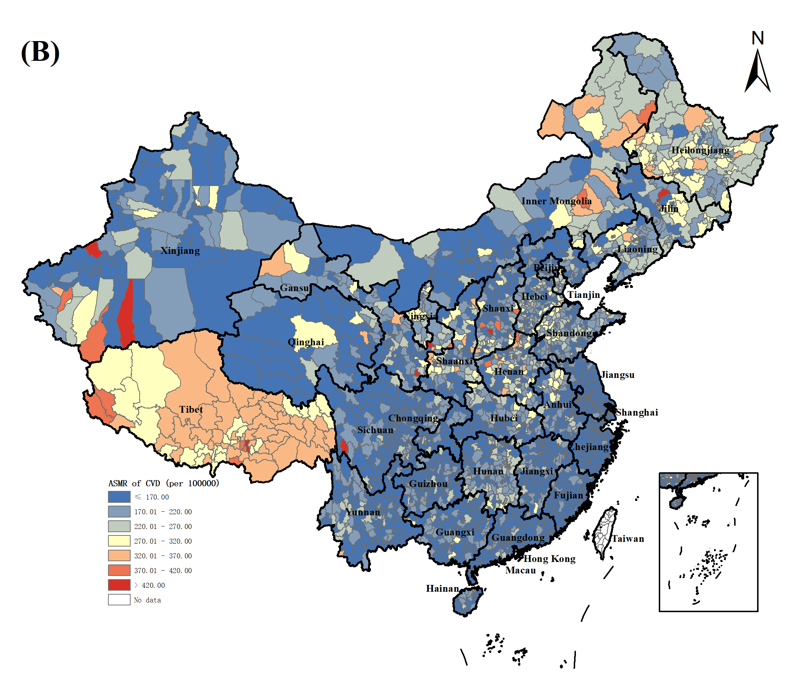

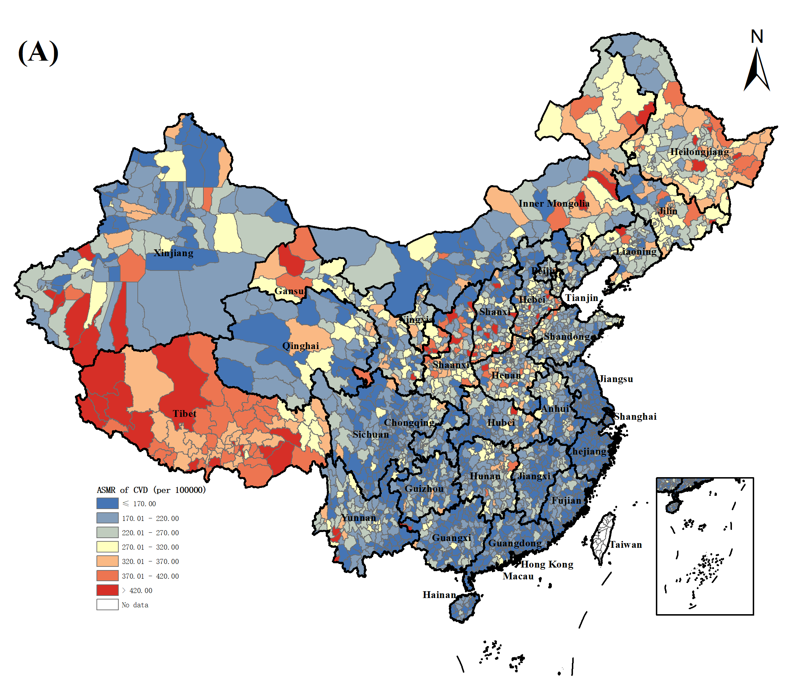


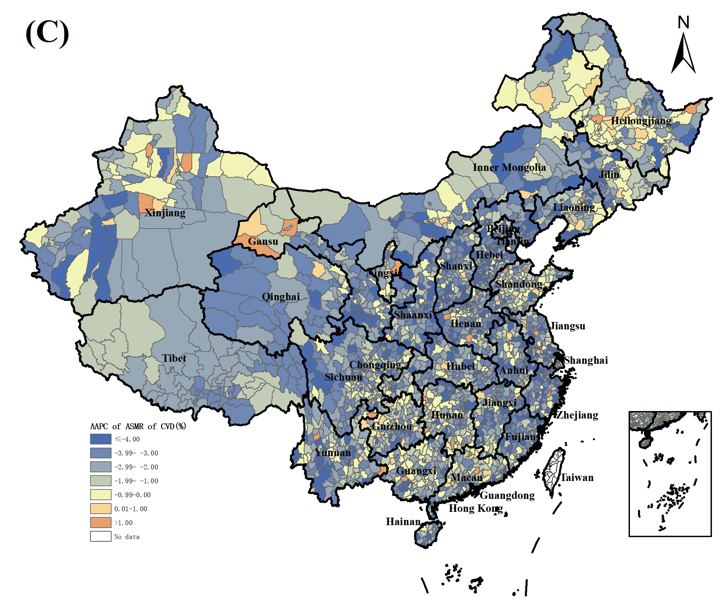


#### Figure S2. ASMR of CVD and its change at county-level in China, female, 2006-2020

(A) ASMR of CVD in 2006, per 100000

(B) ASMR of CVD in 2020, per 100000

(C) AAPC of ASMR of CVD during 2006-2020 (%)

**Footnotes:** ASMR: age-standardized mortality rate; CVD: cardiovascular disease; AAPC: annual average percent change

### 4. Spatiotemporal patterns of relative risk of CVD mortality at county-level in China, by sex, 2006-2020


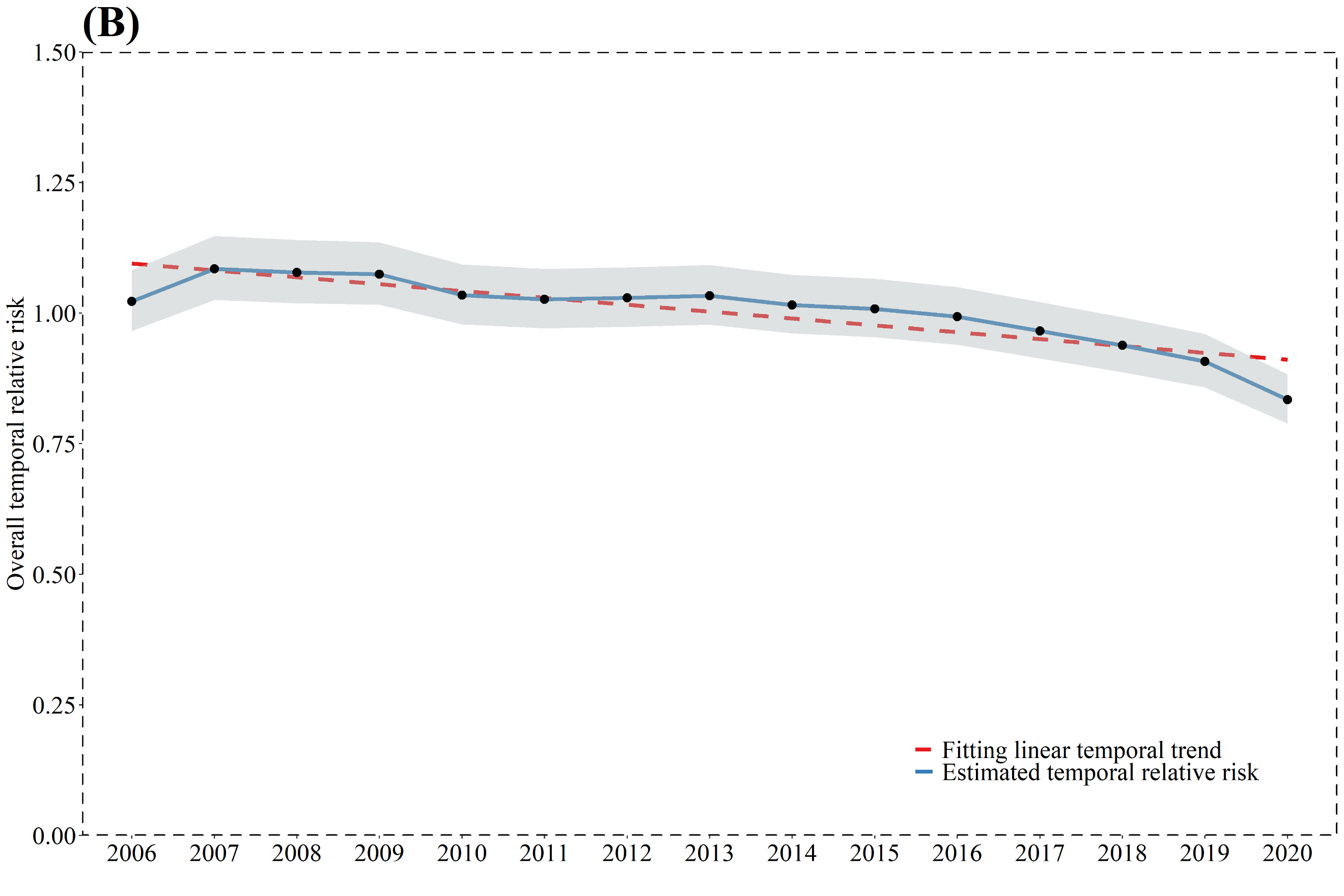

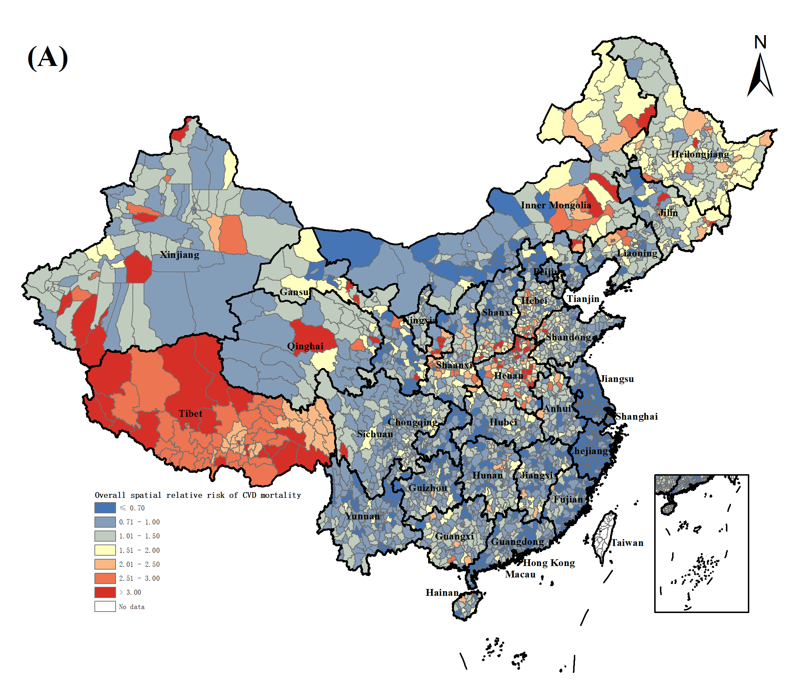


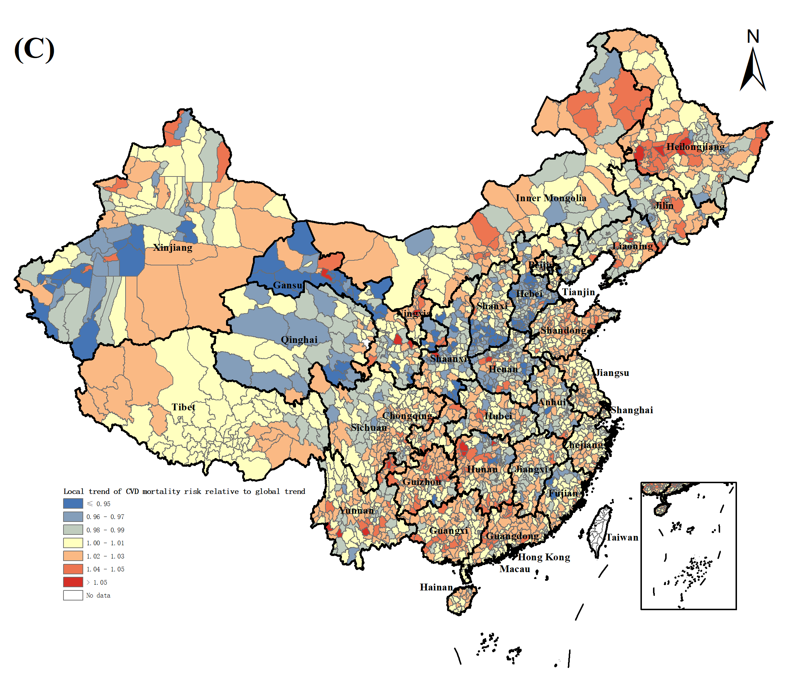


#### Figure S3. Spatiotemporal patterns of relative risk of CVD mortality at county-level in China, male, 2006-2020

(A) Posterior mean of the overall spatial relative risk $(exp\left( s_{i}+u_{i} \right))$of CVD mortality, male, 2006-2020

(B) Posterior mean of the overall temporal relative risk $(exp\left( b_{0}t+v_{t} \right))$ with probability of 95% of CVD mortality, male, 2006-2020

(C) Posterior mean of the local spatiotemporal trend $(exp\left( b_{1i} \right))$of CVD mortality relative to global spatiotemporal trend, male, 2006-2020

**Footnotes:** ASMR: age-standardized mortality rate; CVD: cardiovascular disease


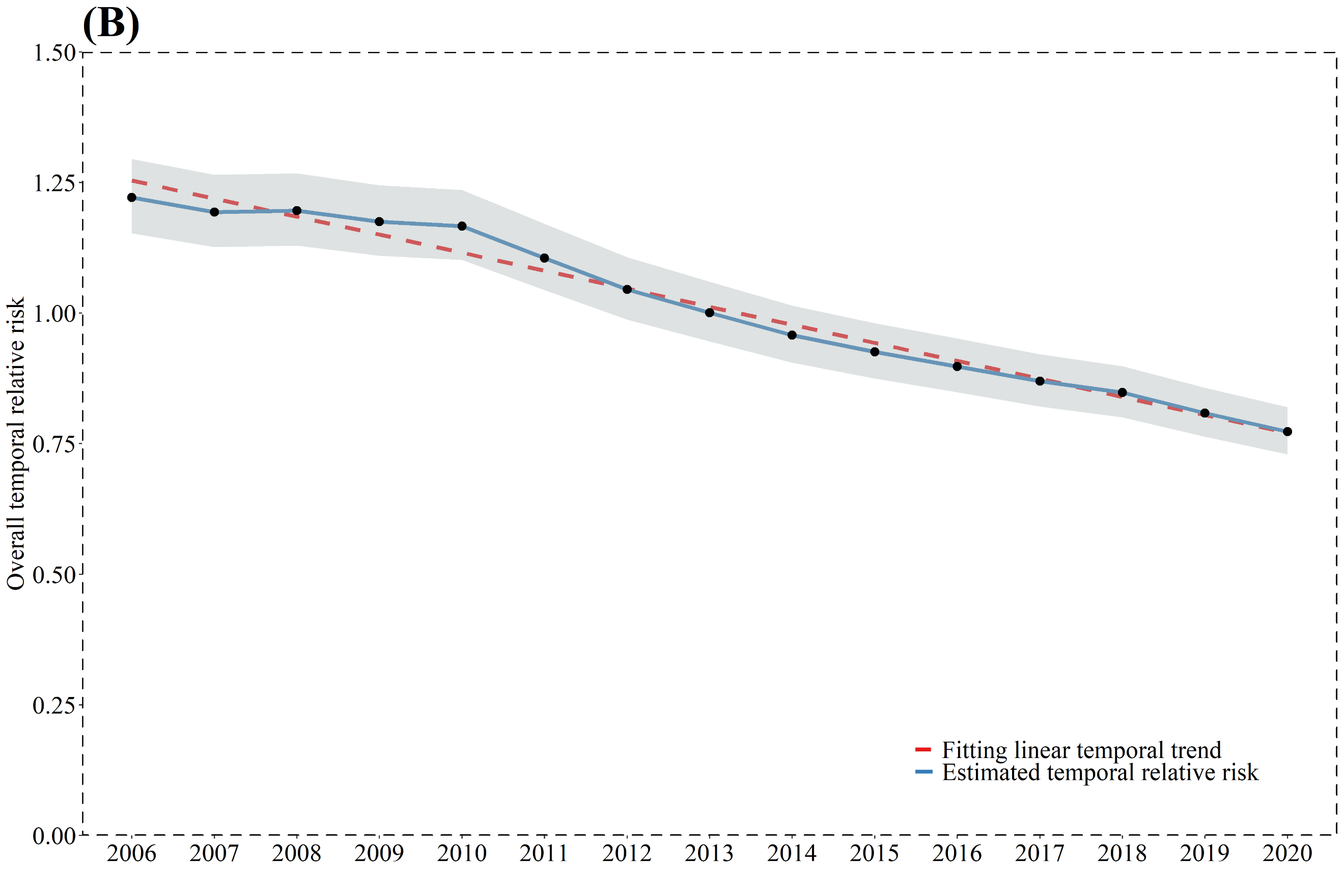

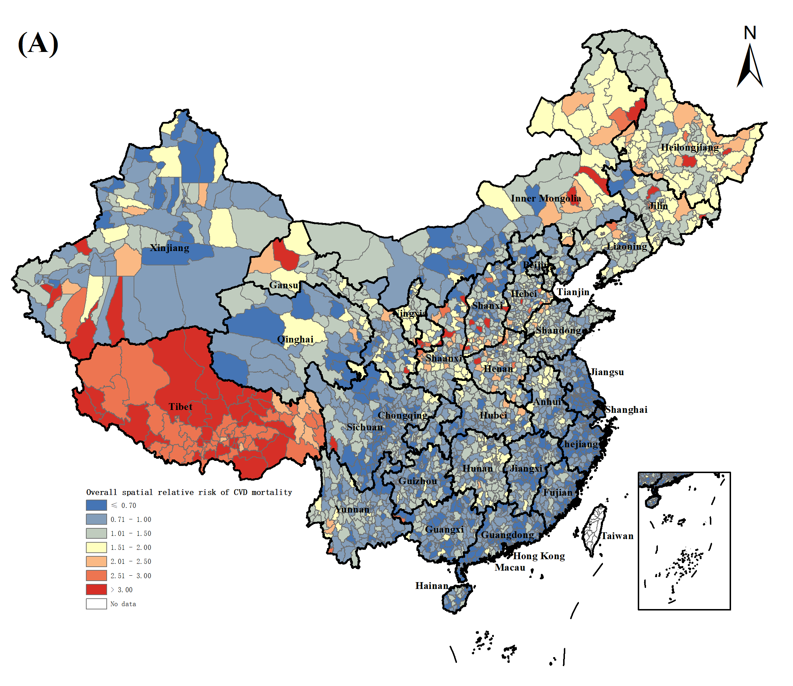


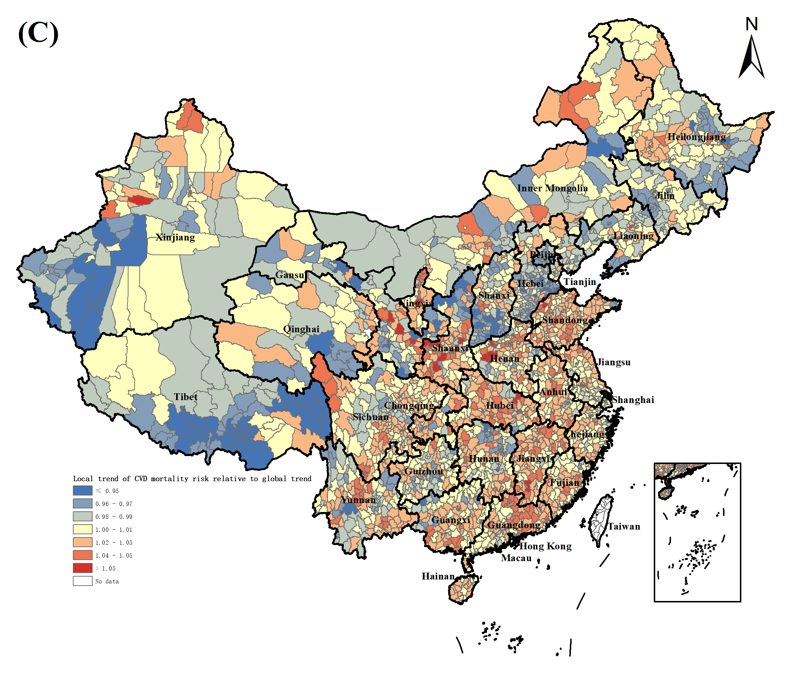


#### Figure S4. Spatiotemporal patterns of relative risk of CVD mortality at county-level in China, female, 2006-2020

(A) Posterior mean of the overall spatial relative risk $(exp\left( s_{i}+u_{i} \right))$of CVD mortality, female, 2006-2020

(B) Posterior mean of the overall temporal relative risk $(exp\left( b_{0}t+v_{t} \right))$ with a probability of 95% of CVD mortality, female, 2006-2020

(C) Posterior mean of the local spatiotemporal trend $(exp\left( b_{1i} \right))$of CVD mortality relative to global spatiotemporal trend, male, 2006-2020

**Footnotes:** ASMR: age-standardized mortality rate; CVD: cardiovascular disease

#### Table S8. Cross classification of spatiotemporal relative risk for CVD mortality, by sex (N, %) ^*^

| **Temporal trends**  **Spatial pattern** | **Stronger local trend compared with global trend** | **Weaker local trend compared with global trend** | **Approximate local trend compared with global trend** | **Total** |
| --- | --- | --- | --- | --- |
| **Hot spots** | Male: 402 (39.37%)  Female: 316 (31.99%) | Male: 347 (33.99%)  Female: 346 (35.02%) | Male: 272 (26.64%)  Female: 326 (33.00%) | Male: 1021 (100%)  Female: 988 (100%) |
| **Cold spots** | Male: 533 (40.32%)  Female: 535 (41.38%) | Male: 361 (27.31%)  Female: 314 (24.28%) | Male: 428 (32.38%)  Female: 444 (34.34%) | Male: 1322 (100%)  Female: 1293 (100%) |
| **Warm spots** | Male: 236 (38.00%)  Female: 289 (42.31%) | Male: 190 (30.60%)  Female: 177 (25.92%) | Male: 195 (31.40%)  Female: 217 (31.77%) | Male: 621 (100%)  Female: 683 (100%) |

**Footnotes:**

^*^N represented to the number of counties that were classified to each of the categories according to the posterior probability estimated by HBSTM during 2-stage classification. The symbol % represented to the percentage of certain number of counties in each of the classification occupied total counties in China.
